# The relationship between social order and crime in Nottingham, England

**DOI:** 10.1038/s44284-024-00161-2

**Published:** 2024-11-01

**Authors:** Federico Varese, Fanqi Zeng

**Affiliations:** 1 Centre d’études européennes et de politique comparée, Sciences Po, Paris, France; 2 Nuffield College, University of Oxford, Oxford, UK; 3 Department of Sociology, University of Oxford, Oxford, UK

## Abstract

Studies of organized crime in cities have traditionally concentrated on the global south or ethnic enclaves and traditional mafia territories within the global north. Here this study turns its attention to governance-type organized crime in the English city of Nottingham, where the main protagonists are white and British born. It investigates whether such a gang can govern communities by reducing ordinary crimes. We conduct in-depth interviews with local officials and analyze a novel dataset of the public’s phone calls to the Nottingham police from 2012 to 2019, encompassing spatio-temporal information and police-labeled crime types. We identify Nottingham’s ward of Bestwood as the site of an entrenched, governance-type organized crime group, whereas its most similar ward, Bulwell, is not. Further comparative analyses indicate that certain ordinary crime rates are significantly lower in Bestwood than in Bulwell. We conclude that governance-type organized crime can emerge in a country with a high capacity to police and in a nonimmigrant, less affluent community. Our findings suggest that traditional explanations of the emergence of criminal governance in cities need to be revisited.

Organized crime groups (OCGs) can engage in a variety of activities in cities and beyond, such as producing illegal goods and services and trading across localities^1^. For instance, cocaine is produced in Colombia and shipped to Europe^2^. However, some OCGs do more: they exercise a degree of governance over communities by imposing restrictions on people’s behavior, enforcing agreements, solving disputes and imposing a rough kind of justice, taking over state functions^3–5^. Research on such governance-type organized crime dates back to the work of Landesco^6^, and notable contributions include studies on traditional mafias^7–12^, urban gangs in the USA^3–19^ and cartels and ‘combos’ in Latin America^20–30^, as well as recent work in the UK^31,32^. In all cases studied in the literature, criminal governance, to some extent, coexists with state governance, a feature that sets it apart from governance by rebel groups. However, most literature focuses on Latin America, traditional mafia territories or immigrant communities in urban settings. The two assumptions, either explicit or implicit, are that such groups operate in a context of states that are ‘infrastructurally weak’^29^ or in immigrant communities that cannot turn to legitimate institutions.

In this study, we focus on an English city in the global north, that is, Nottingham. The core aim of this paper is to establish whether a local criminal group has been able to govern one community by reducing the level of ordinary crime and punish social deviants^8,9^. In doing so, we also map crime in the city. We exploit a novel dataset comprising the public’s phone call records to the police in Nottingham from 2012 to 2019. This dataset includes time, location details and crime types (CTs) labeled by law enforcement, enabling us to undertake a macro-level spatial analysis of eight major crimes over time. Initially, we placed postcode-level phone call data into the smallest output area (lower-layer super output areas (LSOAs)) made available by the census in England and Wales^33^, and then we aggregated the data into Nottingham’s 20 wards. We used spatial and correspondence analysis techniques to unveil associations between wards and crime categories. Our findings underscore an uneven distribution of specific crimes within Nottingham, with particular offenses being concentrated in specific areas of the city. Furthermore, our macro-level analysis suggests the persistence of certain crimes in Nottingham over time.

Alongside the quantitative analysis, we conducted field interviews with local police officers. We also analyzed books and news items related to crimes in Nottingham and undertook field trips to evaluate the neighborhoods’ urban buildup. The ward of Bestwood, a working-class community without many immigrants^33–35^, emerges as the area where a governance-type OCG, led by its boss, Colin Gunn, has been active for decades^34^. Through a matching comparison analysis, we identified Bulwell as a ward sharing comparable socioeconomic and demographic profiles with Bestwood, yet diverging concerning the presence of a governance-type OCG. We then show that where the governance OCG is present, certain crime rates are lower. Thus, Bulwell can be considered a reasonable counterfactual for what Bestwood would have looked like without Gunn’s gang.

This study combines qualitative insights and quantitative analyses, setting it apart from most works on organized crime in cities. Many of the classic studies are qualitative, while others analyze large-scale data. Our study follows in the footsteps of a small but growing set of papers that combines ethnographic insights with large-scale data collection and analysis (for example, refs. 27,28,30,36). In our study, the field interviews helped us identify the wards to study with large data. Ultimately, we show that a governance-type OCG exists in a nonimmigrant and relatively poor community in an English city, where the state is neither weak nor emerging from many years of civil war or in the proximity of global drug cartels^28,37^. While further research is needed to identify local causes of the emergence of criminal governance, traditional explanations do not suffice.

## Results

### Yearly criminal patterns were similar in the same ward

**Fig. 1:**
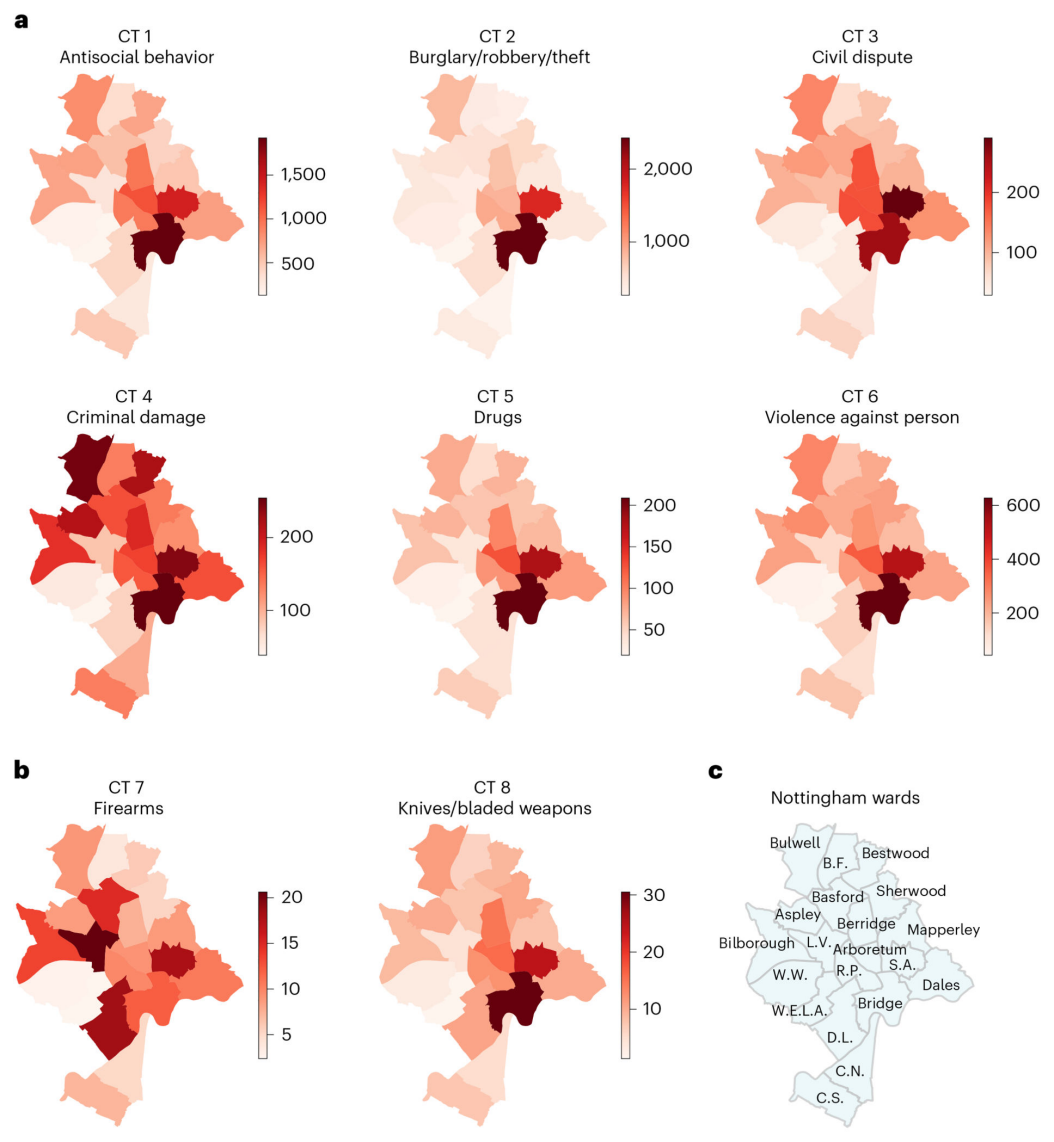
Maps of the average crime counts in the 20 wards of Nottingham (2012–2019). Each ward is colored based on its average crime count during the period. Deep red represents a high crime volume, and the color bars indicate the average counts of associated CTs. **a**, Maps showing the six CTs (CT 1–CT 6) that are mainly against people. **b**, Maps showing the two CTs (CT 7 and CT 8) that are mainly associated with the possession of tools involved in criminal activities. These are notably less than the six crimes in **a** in terms of volume. **c**, The annotated wards map of Nottingham City. Here, we use the pre-2019 Nottingham City Ward map^54^ for the mapping. Some names of wards are abbreviated, that is, Bulwell Forest (B.F.), Clifton North (C.N.), Clifton South (C.S.), Dunkirk and Lenton (D.L.), Lean Valley (L.V.), Radford and Park (R.P.), St Ann’s (S.A.), Wollaton West (W.W.) and Wollaton East and Lenton Abbey (W.E.L.A.). Large Nottingham wards map can also be found in ref. 54.

We first investigated the average crime counts of eight CTs in the 20 wards of Nottingham city during 2012–2019, as mapped in Fig. 1 (see Supplementary Table 1 for details). Some macro patterns emerge. For instance, Bridge and St Ann’s—in the center of the city—attract high levels of all types of crime. This is where shopping centers, restaurants and malls are predominantly located. As we shall see below, this an area of ‘disorganized’ and highly violent crime. Neighborhoods in the periphery of the city, both south and north, generally experience lower levels of crime, yet there are differences. Lean Valley has a high level of firearms crime, whereas Bulwell, not Bestwood, in the north of the city, ranks higher for criminal damage. The overall crime trends in the 182 LSOAs of Nottingham City did not change greatly during the 2012–2019 period (Supplementary Fig. 1).

### Field interviews: the Bestwood governance-type OCG

Our analysis of news items, a book-length journalistic account of criminality in the city and 13 interviews with police officers uncovered the existence of a governance-type OCG entrenched in the neighborhood of Bestwood, in the northeast of the city. Its leader is convicted murderer Colin Gunn who, together with his ‘Bestwood Cartel’ gang, is linked to several murders, police corruption and large-scale drugs trafficking in the Nottingham area^38,39^. Together with his brother David and others, he has been active in Bestwood since the 1980s^39,40^. The gang “by the late nineties, [was] running protection rackets all over Bestwood”,^34^ in a context where there was deep distrust of the police and notable drugs distribution (officers 10 and 11). Colin even positioned people watching cars coming in and out of the Bestwood police car park (officer 10). The gang was perceived to be “the real authority in the community”, to which residents would turn to have a wrong redressed, as detailed in a book-length investigation^34^. “In Bestwood, [Colin] was God-like”, officer 2 told us, a view shared by Pat Chambers, manager of the community center on the Bestwood Estate, who said “He was the boss, the mafia boss. If you have a problem, you did not go to the police, you went to Colin, and Colin sorted it. […] Colin helped people and people loved him.”^41^. When Colin’s nephew died, he expected residents to put obituaries in the local paper to the point that the newspaper had to hire extra staff. Bestwood came to a standstill to allow a horse-drawn cart to go through the town center (officer 10). Colin’s house was “almost like a castle on the hill, in the middle of the estate”, for all to see (officer 10) and he cultivated an image of a local Robin Hood. “I think he probably enjoyed that [role] more than supplying drugs, he loved being that local governance within the community, the person that people looked up to and feared,” (officer 10 and see also ref. 34). Colin and his peers were capable of acts of generosity toward members of the community who were struggling financially and paid for firework displays and raffle prizes for the local schools (officer 10 and ref. 34, “he also lent money”). In return for his generosity, Colin demanded loyalty and asked for favors.

The gang policed ordinary crime. An officer who has worked in Bestwood in the 1990s and 2000s recalls that, in his time on the estate, “you didn’t commit burglaries or car thefts or the like on the Bestwood Estate because Colin would police that himself and he was more effective in dealing with it than the police,” (officer 10). For instance, “a local teacher got her car broken into and, by the time the police got there, it’s been resolved by Colin, ‘no, I’ve got my property. Colin sorted that out I don’t need you’, she told me,” (officer 10). A particular type of crime committed by the gang, according to our informants, is ‘criminal damage.’ “This is a warning sent by the OCG to a person who is out of line. Criminal damage includes arson attacks, although arson is not very common. It would be more common to smash a window or damaging a car, and it is a form of intimidation.” (officer 10).

Colin Gunn was arrested in 2006^40^. Does he still manage to to exert control over the neighborhood? While the arrest surely reduces his power, his gang continues to be a presence and his reputation still mattered according to interviews. Petty criminals still use his name to increase their standing in a dispute. “You still get people saying ‘well, I’m—you know, Colin Gunn’s second cousin’s, fourth removed former roommate’” (officer 2, see also officers 3, 8 and 10). Colin still manages to communicate from prison with his lieutenants (officers 10 and 11) and to punish those who disrespect him or his family (officer 11 and see also ref. 42). After the arrest, “he absolutely still has got influence” (officer 11 and see also ref. 40).

Police patrolling City Central and City South report high level of crime, but the gangs there are not long lived, often do not have a name and do not engage in the kind of community involvement seen in Bestwood, nor do they have the ability to corrupt public officials, reduce antisocial behavior, settle business disputes or run protection rackets (officers 1–9, 12 and 13). “There is no Robin Hood,” officer 3 told us. In neighborhoods such as St Ann’s and Meadows (Bridge) officers encounter “urban street gangs”, dealing in drugs and engaging in robberies (officers 1 and 2). Prostitution is linked to consumption of illegal substance. Corrupting police officers and local officials is “an echelon too high for them” (officer 2). Officers also note that, in community meetings, local urban gangs are not discussed as a major concern, suggesting that their impact is low (for example, officer 1).

### Correspondence analysis on wards and crimes

**Fig. 2:**
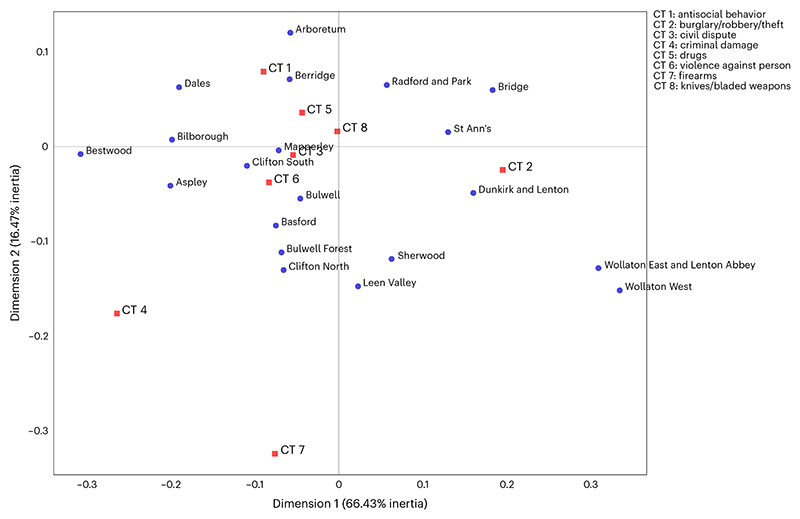
Correspondence analysis of CTs and neighborhoods in Nottingham. The relationships between the two categorical data, that is, 20 wards (blue dots) and 8 CTs (red squares) in two-dimensional space. The associations between variables are illustrated by their closeness in the plot. Dimensions 1 and 2 of the biplot correspondence graph account for 82.9% of the explained inertia, which indicates that the two principal dimensions together can cover the majority association of the categorical data^67^.

Multiple correspondence analysis (MCA) is a model-free technique used to establish the strength between categorical variables and represent them in a geometrical space^43,44^. We apply this method to represent the relationships between CTs and neighborhoods. It transforms rows and columns of a data matrix into points of a two-dimensional Euclidean space^45^ and places categories that are associated close to each other. No pre-existing model is needed, but rather patterns emerge inductively. The existence and the strength of a relationship between two categorical variables can be established by drawing a line from the points of interest to the origin of the principal axes. For instance, in Fig. 2, an imaginary line can be drawn from the point representing Bestwood and the origin, and another imaginary line between CT 4 (criminal damage) and the origin. The angle between the two lines is acute, thus the relationship between the two variables is positive (the scalar product is positive). We can then report that criminal damage is present markedly in Bestwood, relative to all other crimes and places in the same space. If the angle is a right angle, it would mean that there is no relationship (the scalar product between the two vectors is zero) and if the angle is obtuse, the relationship would be negative (the scalar product is negative). The longer the line between the point and the origin, the stronger the relationship^44,45^.

Figure 2 also shows that a large number of neighborhoods are clustered in the center of the plot, together with most crimes, which means these CTs and wards are associated or similar. At the extreme right in Fig. 2 are the suburbs, that is, Wollanton West and Wollanton East and Lenton Abbey, staying far away from most CTs, which indicates that they are similar to each other regarding eight CTs and have low level of crime (this can also be found from their average crime patterns shown in Fig. 1 or yearly crime patterns shown in Supplementary Fig. 2). In sum, Fig. 2 illustrates the relationship between eight CTs and 20 neighborhoods in Nottingham, as well as individual relationships within the same category.

### Matching Bestwood

The MCA helps to place Bestwood in relation to other wards’ crimes at a macro level and, along with the interviews, it raises a further question of what makes Bestwood so special. To answer this question, we select critical socioeconomic features (Table 1) from the 2011 Census for England and Wales^33^ and the English Indices of Deprivation 2015^35^, which have been used in a previous study^46^. We identify the ward most similar to Bestwood and then compare the crime patterns from that ward with Bestwood.

**Fig. 3:**
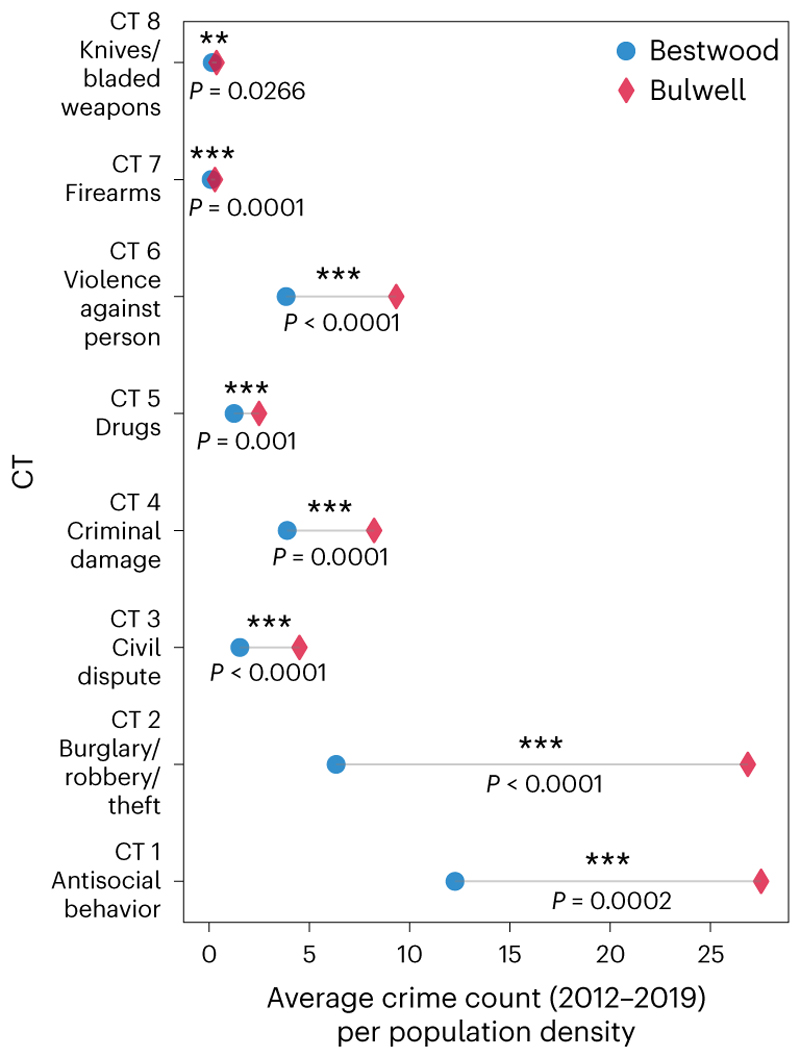
Matching comparison between Bestwood and Bulwell on eight CTs measuring by average crime counts over local population densities. The gray bars illustrate the differences in average crime rates for each CT between the two wards. Overall, Bestwood demonstrates lower average crime counts per population density than Bulwell across all eight CTs, with minimal discrepancies observed for firearms (CT 7) and knives/bladed weapons (CT 8). The *P* values under each bar indicate that, according to two-sided Welch’s *t*-tests (Supplementary Fig. 3), Bestwood’s crime counts per population density are significantly lower than those of Bulwell for each CT over the period. ***P < 0.01 and **P < 0.05.

By applying a nonparametric supervised learning method called *k*-nearest neighbors (*k-*NN), we find Bulwell to be the nearest ward to Bestwood in relation to these socioeconomic features. Figure 3 shows that Bestwood has a lower level of crime than Bulwell on all CTs when measuring by the average crime counts over local population densities. Furthermore, we conducted Welch’s *t*-tests on each CT (Supplementary Fig. 3) for the observed measurements from the two wards over 8 years with 10,000 bootstraps. The statistical analyses, using the crime counts per population density, indicate that Bestwood is significantly lower than its matched ward of Bulwell on every CT.

These quantitative results resonate with the interviews, that is, Colin’s governance-type OCG has major impacts on local crimes. In particular, policing antisocial behavior (CT 1) and burglary/robbery/theft (CT 2) appears to be a notable activity of the gang, which in some sense is in striking parallel to the same activity undertaken by republican armed groups in Northern Ireland^3^. Furthermore, crimes related to the protection of businesses (who are likely to pay protection money), such as burglary/robbery/theft (CT 2), civil dispute (CT 3) and criminal damage (CT 4) are significantly lower in Bestwood than in Bulwell. Crime related to personal safety, such as violence against person (CT 6), is also significantly lower in Bestwood. Crimes, such as drugs (CT 5), firearms (CT 7) and knives/bladed weapons (CT 8), are at approximately the same levels, suggesting that the Bestwood gang does not prevent such activities, which are a form of business and is likely to generate revenues for the gang. However, Fig. 3 shows Bestwood still has significantly lower levels than Bulwell of these three CTs.

On the basis of the yearly measurements in Supplementary Fig. 3, we can see than the ability of the gang in policing crime did not decrease following the arrest of Colin Gunn. As also suggested by the interviews, the grip of the Bestwood gang on the ward does not change much over the years, with the values for Bestwood remaining well below Bulwell, even in later years.

## Discussion

This work applies a mixed-methods approach that contextualizes a large dataset of calls to the police by the public and official census and deprivation index data, as well as qualitative data collected from interviews with local police officers in Nottingham. We first show that crime patterns in the city differ: in the south and central neighborhoods, crimes are quite high, especially in St Ann’s and Bridge, where the main shopping area is located. Gangs converge there to supply drugs and commit robberies. Neighborhoods in the west of the city are suburbs; hence, we see lower crime. The north of the city is the location of less affluent areas, and we observe a lower level of crime than in the center but higher than in the suburbs, and yet there is variation within the north of the city.

Through a series of in-depth interviews, analysis of written documents and field trips, we identify Bestwood as a part of the city where a governance-type OCG operates. Although after the arrest of the main boss, Colin Gunn, the power of the gang has been reduced, the OCG is still exercising governance over the neighborhood. By conducting a matched comparison, we identified Bulwell as the most similar area to Bestwood in the city in terms of several key sociodemographic variables. When examining the distribution of the crime counts per population density, we saw that all eight selected crimes are significantly lower in Bestwood than in Bulwell. Other forms of crime are very similar (drugs, firearms and knives/blades weapons), but very low in volume. Despite the presence of a serious OCG in Bestwood, no serious violence takes place. The quantitative findings resonate with the information we obtained from interviews. We conclude that the gang in Bestwood can reduce expressive crime and crimes that affect the business community, such as antisocial behavior, burglary/robbery/theft, civil disputes, criminal damage and violence against the person. As Bestwood is most similar to Bulwell except for the presence of Gunn’s gang, our findings are consistent with the theory^36,47^ that the gang’s governance activities have reduced crime rates.

Actual crime occurrences may differ from the data documented in official crime statistics, which, in turn, differ from calls reported to police. Local populations might perceive governance-type OCG as trustworthy mediators adept at resolving conflicts and rectifying injustices. Consequently, individuals may reach out to the gang rather than the police. This implication would be that while calls to the police in Bestwood are lower, the actual crime occurrences might not be. Yet scholars should not dismiss anonymous phone lines where crimes can be reported. Such a method has been successfully adopted by several cities, including Ciudad Juárez^48^, one of the most violent cities in Mexico, as well as Rio de Janeiro^49,50^ in Brazil and cities in Guatemala^51^. It should also be noted that the CTs selected for this study do not occur behind closed doors, such as domestic abuse, and often trigger the involvement of insurance companies or even medical attention. Hence, they are more visible and are more likely to generate a response. In addition, we find that reports that are very likely to trigger a call, such as drugs, firearms and knives/bladed weapons, are almost identical in both Bestwood and Bulwell. We interpret this finding as suggesting that individuals in both areas are not deterred from calling the police as these calls are anonymous, such that what we find is a genuine ability of the gang to reduce ordinary crime. It is highly unlikely that the gang has the ability to generate such fear in the community to scare off reporting to the police phone line.

While it is hard to establish causality with observational phone call data, we believe that the matched comparison goes in that direction. As for the risk of reverse causality, there are no specific reasons why this OCG would emerge within communities with low-volume crime or why it would migrate there. Indeed, Colin Gunn grew up in Bestwood^39^.

The key contribution of this work is to extend the study of criminal governance to a city in the global north that is not associated with traditional mafias, drug production or immigrant communities. Bestwood has only 11.4% of residents born outside the UK (Table 1), among the lowest figure in the city (cf. the value for St Ann’s of 46.8%). The ward also has a relatively high level of income and employment deprivation, and residents experience high barriers to reaching social services, are poorly educated and suffer poor health (Table 1). However, Bestwood is not the only ward with such a low income and difficulty accessing social services. Our findings present an urgent call to improve the traditional explanations of criminal governance in cities.

A powerful gang led by a charismatic leader^34^ thrived in a less affluent neighborhood with low immigration. Residents suggested to us that a lack of public facilities, such as parks, social clubs and venues for recreation, fostered the rise of gang culture in a local context where drug profits were high^34^ and state policing wanting. High population density is also a feature that makes Bestwood special. Recent developments in the neighborhood indicate that local authorities have increased social services and engaged in community policing through ‘Operation Reacher’, leading to a reduction of the gang’s activity^52^.

## Methods

### Ethic approval

This research follows all relevant ethical regulations. Approval for the study was granted by the Central University Research Ethics Committee, University of Oxford (R70851/RE004). We have obtained informed consent from all interviewees before the interviews.

### Materials

#### Crime data and socioeconomic data

We use an anonymous dataset of the public’s phone calls to the police in the city of Nottingham from 2012 to 2019 containing call time, postcode-level location and police-labeled CTs. Since the crime data are distributed at the postcode level, we first aggregate every year’s data into LSOAs, which are officially designed areas in England and Wales comprising between 400 and 1,200 households and usually have a resident population between 1,000 and 3,000 (ref. 53). There are 182 LSOAs in Nottingham, making up to 20 wards. We then compute the crime counts in the 20 wards. Notably, from May 2019, Nottingham City Council adjusted the boundaries of several wards except for Bestwood, Bulwell, Bulwell Forest and Wollaton West^54^. Furthermore, the names of a few wards in Nottingham city central and south have been changed since then. For example, the majority area of ward Bridge is now in ward Meadows. Here, considering the crime data we examined, we use the prechange geographic areas and wards map to conduct the analysis. We recognize that these adjustments of ward boundaries would not substantially impact the main results in this work.

We also use the 2011 UK Census data^33^ and the English indices of deprivation data^35^ for Nottingham to characterize the socioeconomic features of neighborhoods. The census and deprivation data are formatted at the LSOA level. Hence we aggregate them into ward level when we make the matching comparison. We select the critical socioeconomic variables, which have been used in a recent criminology study in London, as drivers in analyzing the causes of urban crime^46^.

#### Interviews

To identify the wards with the presence of organized crime in Nottingham, we conducted 13 interviews with senior police officers tasked with the fight against organized crime in the city now and in the recent past. The interviews took place in 2022 and 2023 (see Supplementary Information for more information on the interview times and key points).

### Analyses

#### Selecting eight CTs

Protection theory has been used extensively to study governance-type OCGs. It posits that such groups impose protection payments to businesses in exchange for a safer environment to conduct commercial activities (enforcement of agreements/recovery of debts/protection against theft)^8,36,47,55^. Hence, we expect that civil disputes and burglary-robbery/theft should be lower in areas controlled by such groups as opposed to areas where such groups do not operate. The same theory also posits that governance-type OCGs enforce protection against expressive violent crimes to increase a process of social consensus^36^, hence we expect crimes such as battery and antisocial behavior to be lower. Thus, based on this theory, we selected eight CTs. Some speak to the protection of businesses (burglary/robbery/theft, civil dispute and criminal damage), while other capture the protection against expressive violent crimes (antisocial behavior and violence against person). In addition, we select drugs, a crime that refers to a business activity that might be protected by the governance-type OCG and is very prevalent in the city. Finally, we selected crimes that speak to the means of committing the offense (firearms and knives/bladed weapons). Owing to their nature, these are crimes that are more likely to be reported accurately, hence we can have a sense of whether there is variation in propensity to report depending on the neighborhood (that in turn might be caused by fear).

#### Correspondence analysis

MCA is a multivariate statistical technique^43,44^, conceptually akin to principal component analysis, providing a summary of categorical data in a two-dimensional space. Given two groups of variables, the method can reveal the relative relationships between and within them. In a biplot correspondence graph, variables from different categories are associated when their positions are close to each other in the plot, while variables from the same category are said to be similar if they are nearby. We explore the relative relationships between 20 wards of Nottingham city and eight CTs. We use the two dimensions with the highest variances captured for visualization, as illustrated in Fig. 2. Here, we used the Python library Prince (version 0.13.0)^56^ to conduct the correspondence analysis.

#### Matching comparison

Matching comparison is a statistical method employed in observational and experimental studies to compare two or more groups that are matched or paired based on certain features. The general process is to identify subjects with similar features and then form pairs, wherein one individual from each pair gets one treatment while the other gets a different treatment or acts as a control. We applied the *k-*NN matching approach, thus we can isolate the effects of treatment or intervention being studied while controlling for the impacts of other variables, which enhances the validity of the comparison analysis and the conclusions drawn.

To conduct the comparison, we include nine socioeconomic features on ‘population density (number of persons per hectare)’, ‘population aged 15–24 years (%)’, ‘highest level of qualification: level 4 qualifications and above’, ‘born outside the UK (%)’ and ‘social rented (%)’, which are extracted from the UK Census 2011 data, and ‘income deprivation domain’, ‘employment deprivation domain’, ‘health deprivation and disability domain’ and ‘barriers to housing and services domain’, which are extracted from the Index of Multiple Deprivation. A detailed description of these features can be found in Supplementary Table 2.

We used the matching comparison formula to investigate the role of OCG’s governance in crime concentration. On the basis of our interviews, we know that the ward of Bestwood has a governance-type OCG existing from at least the 1980s. We wanted to establish whether any other ward in Nottingham with similar socioeconomic status to Bestwood but without a similar type of OCG displays different crime patterns.

#### *k-*NN

The *k-*NN algorithm is a nonparametric supervised learning method that is widely used in classification or regression^57,58^. Here, we use the Python library Scikit-learn (version 1.3.2)^59^ to find the nearest neighbor in terms of socioeconomic features for Bestwood. We first scaled the socioeconomic features by the maximum values in each feature respectively to ensure all features are on roughly the same scale of (−1, 1). Then, we use the Minkowski metric $d(x,y)={\left(\Sigma_{i=1}^{n}|x_{i}-y_{i}{|}^{p}\right)}^{1/p}$ to compute the similarity distance *d*(*x, y*) between ward *x* and ward *y*, wherein *x*_*i*_ (here *i* = 1, …, 9) represents the socioeconomic features of ward *x* and *p* is the parameter for the Minkowski metric in the Scikit-learn package. When *p* = 1, the *k-*NN algorithm in the Scikit-learn package is equivalent to using the Manhattan distance $L^{1}=\Sigma_{i=1}^{n}|x_{i}-y_{i}|$, and *p* = 2 for the Euclidean distance $L^{2}=\sqrt{\Sigma_{i=1}^{n}{\left(x_{i}-y_{i}\right)}^{2}}$. We check both *p* = 1 and *p* = 2, and it turns out that Bulwell is the nearest ward to Bestwood in both situations (Supplementary Table 4). Therefore, we conclude that Bulwell is the most similar ward to Bestwood in terms of the critical socioeconomic status that has a relationship with crime.

#### Statistical significance and robustness check

When finding the nearest ward to Bestwood, we used the above-mentioned two similarity metrics as a robustness check, which both indicate Bulwell is the most similar neighborhood to Bestwood. After obtaining the matched pair of Bestwood and Bulwell, we conducted a Welch’s *t*-test (two sided)^60^, along with bootstrapping (10,000 times per individual CT) to check whether the two wards are significantly different on individual CTs.

When using the measurement of crime count per population density for the Welch’s *t*-tests, Bestwood was significantly lower than Bulwell on all eight CT measurements (Supplementary Fig. 3). We choose this measurement for two main reasons: first, Bestwood’s population density is almost double that of Bulwell’s and second, population density is often considered to have an impact on crime volume^61,62^. Therefore, a scaled comparison of crime, based on local population densities between two wards, may increase the robustness of the result.

Meanwhile, we used the measurement of crime count to conduct similar statistical analyses. We perform a Chi-squared test using the average crime counts, which shows that Bestwood and Bulwell are significantly different regarding the eight CTs (Supplementary Fig. 4). Furthermore, Supplementary Figs. 4 and 5 show that Bestwood is still significantly lower than Bulwell on CT 2, CT 3, CT 6 and CT 7 when using the measurement of crime count, while Bestwood is lower than Bulwell regarding average crime count on CT 1, CT 4, CT 5 and CT 8, as indicated in Supplementary Fig. 4.

Additionally, we conducted a comparison between Bestwood and its second-most similar neighbor, Bilborough (Supplementary Table 4). As shown in Supplementary Fig. 6, Bestwood is significantly lower than Bilborough on almost every CT except for CT 8 when measured by population density. Additionally, we compared Bestwood with its three neighbors, that is, Basford, Bulwell Forest and Sherwood, which are not very similar to Bestwood regarding socioeconomic features (Supplementary Table 4) and have lower population densities than Bestwood (Table 1). The additional comparisons (see Supplementary Information for details) show that Bestwood is still significantly lower than its neighbors on certain crimes, such as CT 2 and CT 3.

#### Built environment in Bestwood and Bulwell

We visited Nottingham several times to conduct the interviews, collect data from the police and observe the built environment, especially the matched wards of Bestwood and Bulwell. We also visited Clifton South and Nottingham City Center (see photos in Supplementary Information). The neighborhoods of Bestwood and Bulwell are very similar. They have similar detached and semi-detached homes and some basic shops, and the streets are well lit at night. Bulwell has no particular high-profile venues that could attract robbers. Public street touring-style videos from Bestwood^63,64^ and Bulwell^65,66^ also resonate with our observation. We conclude that the built environment of Bestwood and Bulwell could not account for the differences in crime rates.

### Reporting summary

Further information on research design is available in the Nature Portfolio Reporting Summary linked to this article.

## Supplementary Material

Reporting Summary

Supplementary Code

Supplementary Information

## Figures and Tables

**Table 1:** Summary of critical socioeconomic features of the 20 wards in Nottingham

Ward	Population density (number of persons per hectare)	Population aged 15–24years (%)	Highest level of qualification: level 4 qualifications and above	Born outside the UK (%)	Social rented (%)	Income deprivation domain	Employment deprivation domain	Health deprivation and disability domain	Barriers to housing and services domain
Arboretum	76	45.2	19.1	29.3	42.7	0.23	0.17	1.34	34.53
Aspley	62.5	15.8	9.8	14.9	48.3	0.41	0.28	1.14	28.79
Basford	42.8	14.6	18.9	13.2	28.3	0.24	0.18	0.85	22.61
Berridge	76	17.6	30.4	29.5	16.5	0.23	0.17	0.73	22.27
Bestwood	57	14.6	13.3	11.4	40.8	0.29	0.23	1.12	23.57
Bilborough	32.8	13.1	11.6	10.2	44.1	0.33	0.28	1.45	22.70
Bridge	32.4	26.7	32	30.6	31	0.21	0.14	0.97	29.51
Bulwell	30.1	14.2	10	8.2	40.4	0.34	0.26	1.21	28.78
Bulwell Forest	41.1	11.8	16.1	8.8	16.1	0.17	0.15	0.55	17.61
Clifton North	28	17.9	17	8.1	19.5	0.15	0.13	0.57	23.68
Clifton South	32.1	13.3	11.3	6.5	31.4	0.23	0.19	1.00	25.31
Dales	32.5	13.7	20.6	23.7	26.7	0.26	0.21	0.96	20.09
Dunkirk and Lenton	20.9	60.8	26.9	28.6	23.5	0.09	0.06	0.62	30.73
Leen Valley	35.2	13.1	20	25.4	20	0.18	0.15	0.65	23.28
Mapperley	43.9	14.7	34.2	19.9	20	0.19	0.17	0.70	25.33
Radford and Park	88.6	48.7	28	31.4	27.4	0.13	0.10	0.61	32.52
St Ann’s	76.1	32.1	17.6	30	46.8	0.28	0.21	1.35	32.24
Sherwood	48.3	12.6	35	14.5	18.2	0.19	0.16	0.76	16.36
Wollaton East and Lenton Abbey	34.6	58	21.9	27.5	28.5	0.10	0.06	0.40	36.39
Wollaton West	26.6	11.1	39.3	17	10.9	0.08	0.08	-0.34	17.85

## Data Availability

The Shapefile we used to produce the wards map of Nottingham in Fig. 1 was published by the Office for National Statistics as an open data file, that is, Wards (December 2018) Full Extent Boundaries UK (https://www.data.gov.uk/dataset/8b829024-b053-4bdd-8133-d91d6e812008/wards-december-2018-full-extent-boundaries-uk). The UK Census 2011 data are publicly available at https://www.ons.gov.uk/census/2011census. The English indices of deprivation data are also publicly available at https://opendatacommunities.org/data/societal-wellbeing/imd/indices. We have no right to share the anonymous phone call data used in the study. We are happy to share the contact of the data officer at Nottingham Police, who has the authority to share the data. We can share the interview data upon reasonable request.

## Code availability

Custom code was created using Python (version 3.8.8). We also used the Python libraries: csv (version 1.0), geopandas (version 0.13.2), matplotlib (version 3.7.1), numpy (version 1.21.6), pandas (version 1.5.3), Prince (version 0.13.0), seaborn (version 0.11.1), scipy (version 1.11.4) and Scikit-learn (version 1.3.2) for essential analysis. The sample code for bootstrap statistical analysis is provided in Supplementary Code.
