# Supplementary material for: The relationship between social order and crime in Nottingham, England
Source: Nat Cities. Author manuscript; Available in PMC 2025 Jul 23. (PMC7617934; doi:10.1038/s44284-024-00161-2)
Supplement: Reporting Summary [file EMS206553-supplement-Reporting_Summary.pdf]

nature portfolio

Corresponding author(s): Fanqi Zeng

Last updated by author(s): Aug 8, 2024

Reporting Summary

Nature Portfolio wishes to improve the reproducibility of the work that we publish. This form provides structure for consistency and transparency in reporting. For further information on Nature Portfolio policies, see our Editorial Policies and the Editorial Policy Checklist.

Statistics

For all statistical analyses, confirm that the following items are present in the figure legend, table legend, main text, or Methods section.

n/a	Confirmed	
☐	☒	The exact sample size (*n*) for each experimental group/condition, given as a discrete number and unit of measurement
☐	☒	A statement on whether measurements were taken from distinct samples or whether the same sample was measured repeatedly
☐	☒	The statistical test(s) used AND whether they are one- or two-sided<br>*Only common tests should be described solely by name; describe more complex techniques in the Methods section.*
☒	☐	A description of all covariates tested
☐	☒	A description of any assumptions or corrections, such as tests of normality and adjustment for multiple comparisons
☐	☒	A full description of the statistical parameters including central tendency (e.g. means) or other basic estimates (e.g. regression coefficient) AND variation (e.g. standard deviation) or associated estimates of uncertainty (e.g. confidence intervals)
☐	☒	For null hypothesis testing, the test statistic (e.g. *F*, *t*, *r*) with confidence intervals, effect sizes, degrees of freedom and *P* value noted<br>*Give P values as exact values whenever suitable.*
☒	☐	For Bayesian analysis, information on the choice of priors and Markov chain Monte Carlo settings
☒	☐	For hierarchical and complex designs, identification of the appropriate level for tests and full reporting of outcomes
☒	☐	Estimates of effect sizes (e.g. Cohen's *d*, Pearson's *r*), indicating how they were calculated

Our web collection on statistics for biologists contains articles on many of the points above.

Software and code

Policy information about availability of computer code

Data collection	No custom code was used for data collection.
Data analysis	Custom code was created using Python (version 3.8.8). We also used the Python libraries: csv (version 1.0), geopandas (version 0.13.2), matplotlib ((version 3.7.1), numpy (version 1.21.6), pandas (version 1.5.3), Prince (version 0.13.0), seaborn (version 0.11.1), scipy (version 1.11.4), and Scikit-learn (version 1.3.2) for essential analysis. We provide the sample code for bootstrap analysis in the Supplementary Code.

For manuscripts utilizing custom algorithms or software that are central to the research but not yet described in published literature, software must be made available to editors and reviewers. We strongly encourage code deposition in a community repository (e.g. GitHub). See the Nature Portfolio guidelines for submitting code & software for further information.

Data

Policy information about availability of data

All manuscripts must include a data availability statement. This statement should provide the following information, where applicable:

- Accession codes, unique identifiers, or web links for publicly available datasets
- A description of any restrictions on data availability
- For clinical datasets or third party data, please ensure that the statement adheres to our policy

The Shapefile we used to produce the wards map of Nottingham in Figure 1 was published by the Office for National Statistics as open data, i.e., Wards (December 2018) Full Extent Boundaries UK (https://www.data.gov.uk/dataset/8b829024-b053-4bdd-8133-d91d6e812008/wards-december-2018-full-extent-boundaries-uk).

The UK Census 2011 data are publicly available at https://www.ons.gov.uk/census/2011census. The English indices of deprivation data are also publicly available at https://opendatacommunities.org/data/societal-wellbeing/imd/indices. We have no right to share the anonymous phone call data used in the study. We are happy to share the contact of the data officer at Nottingham Police, who has the authority to share the data. We can share the interview data upon reasonable request.

Research involving human participants, their data, or biological material

Policy information about studies with human participants or human data. See also policy information about sex, gender (identity/presentation), and sexual orientation and race, ethnicity and racism.

Reporting on sex and gender	We do not report on the sex and gender of the participants. This work does not analyze any sex or gender information. Regarding the official census and deprivation data, there is no such information on individual human beings. Regarding the anonymized phone call data, there is no such information on individuals either. For the interview participants, we do not consider sex and gender.
Reporting on race, ethnicity, or other socially relevant groupings	We use the socioeconomic variables provided in the official census and deprivation data, which include variables such as the population aged 15-24 (%) and the percentage of individuals born outside the UK. These variables were aggregated at geographic levels and contained no individual information on race or ethnicity. There is no information on race, ethnicity, or other socially relevant groupings in the anonymized phone call data. For the interview participants, we did not consider any variables related to race, ethnicity, or other socially relevant groupings.
Population characteristics	See above.
Recruitment	For the interviews, we first contacted the Nottingham Police Department, which then selected the police officers as interviewees based on their working experience and policing areas in the city. Although the Nottingham Police Department recommended these senior officers, selection bias may exist. For example, some officers might only be familiar with the same area. To reduce this bias, we made every effort to get as many interviewees from different areas as possible. During the interviews, we also asked the interviewees about crime in other city areas, as a cross-check.
Ethics oversight	Approval for the study was granted by the Central University Research Ethics Committee, University of Oxford (R70851/RE004).

Note that full information on the approval of the study protocol must also be provided in the manuscript.

Field-specific reporting

Please select the one below that is the best fit for your research. If you are not sure, read the appropriate sections before making your selection.

☐ Life sciences ☒ Behavioural & social sciences ☐ Ecological, evolutionary & environmental sciences

For a reference copy of the document with all sections, see nature.com/documents/nr-reporting-summary-flat.pdf

Behavioural & social sciences study design

All studies must disclose on these points even when the disclosure is negative.

Study description	This work is a mixed-method case study using quantitative large-scale data analysis and qualitative interviews.
Research sample	Based on official census data, we selected 20 wards (182 LSOAs) of Nottingham City as the research samples to investigate criminal governance in the city. We used police-labeled public phone calls from 2012 to 2019, along with official socio-economic data for these regions, to conduct the study. These datasets cover the entire city of Nottingham, making them representative of the area. In our analysis, we used the aggregated variables of the population aged 15-24 (%) and the % born outside the UK as provided in the census data. There is no demographic information in the anonymized phone call data. Additionally, we conducted 13 interviews with police officers who patrol Nottingham. The Nottingham Police Department selected these 13 interviewees based on their work experience and areas of responsibility, and they recommended the interviewees to us.
Sampling strategy	We analyzed data from the entire city of Nottingham during the observation period (2012-2019) using official sources. We also interviewed 13 senior police officers from different areas of the city who had extensive knowledge of local crime. The Nottingham Police Department selected the interviewees based on their work experience and patrolling areas within the city.
Data collection	The UK Census 2011 and the English indices of deprivation data (2015) were obtained from official public websites, as cited in the work. We received the anonymized phone call data from the Nottingham police department. For the qualitative interview data, we conducted 13 interviews with police officers in Nottingham. The Nottingham Police Department recommended the interviewees.
Timing	The UK Census 2011 and the English indices of deprivation data (2015) were collected and publicly released by the UK government in 2012 and 2015, respectively. We downloaded these data from the official website when we conducted the work in 2022. The phone call data were collected by the Nottingham police from 2012 to 2019. We received the anonymized data in 2022. We interviewed 13 police officers in Nottingham from 2022 to 2023.
Data exclusions	For the crime data we received from the Nottingham Police Department, we selected 8 main crime types and merged the police-labeled types that were closely related to these 8 main categories. We excluded 9 categories of police-labeled data that were

	irrelevant to the 8 main crime types.
Non-participation	No participant dropped out the study.
Randomization	For the qualitative interviews, the interviewees were recommended by the Nottingham Police Department based on the interviewee's working experience and patrolling area, which was not a randomization.

Reporting for specific materials, systems and methods

We require information from authors about some types of materials, experimental systems and methods used in many studies. Here, indicate whether each material, system or method listed is relevant to your study. If you are not sure if a list item applies to your research, read the appropriate section before selecting a response.

Materials & experimental systems

n/a	Involved in the study
☒	☐ Antibodies
☒	☐ Eukaryotic cell lines
☒	☐ Palaeontology and archaeology
☒	☐ Animals and other organisms
☒	☐ Clinical data
☒	☐ Dual use research of concern
☒	☐ Plants

Methods

n/a	Involved in the study
☒	☐ ChIP-seq
☒	☐ Flow cytometry
☒	☐ MRI-based neuroimaging

Plants

Seed stocks	*Report on the source of all seed stocks or other plant material used. If applicable, state the seed stock centre and catalogue number. If plant specimens were collected from the field, describe the collection location, date and sampling procedures.*
Novel plant genotypes	*Describe the methods by which all novel plant genotypes were produced. This includes those generated by transgenic approaches, gene editing, chemical/radiation-based mutagenesis and hybridization. For transgenic lines, describe the transformation method, the number of independent lines analyzed and the generation upon which experiments were performed. For gene-edited lines, describe the editor used, the endogenous sequence targeted for editing, the targeting guide RNA sequence (if applicable) and how the editor was applied.*
Authentication	*Describe any authentication procedures for each seed stock used or novel genotype generated. Describe any experiments used to assess the effect of a mutation and, where applicable, how potential secondary effects (e.g. second site T-DNA insertions, mosiacism, off-target gene editing) were examined.*
