# Supplementary material for: The relationship between social order and crime in Nottingham, England
Source: Nat Cities. Author manuscript; Available in PMC 2025 Jul 23. (PMC7617934; doi:10.1038/s44284-024-00161-2)
Supplement: Supplementary Information [file EMS206553-supplement-Supplementary_Information.pdf]

nature cities

Supplementary information https://doi.org/10.1038/s44284-024-00161-2

The relationship between social order and crime in Nottingham, England

In the format provided by the authors and unedited

Supplementary Information:

Contents

SI.1 General crime trends in Nottingham city from 2012 to 2019 2
SI.2 Selected socioeconomic features 5
SI.3 Field interviews 6
SI.4 Crimes in Bestwood and Bulwell 9
SI.5 Crimes in Bestwood and its second-most similar and nearby wards 13
SI.6 Building environment in Nottingham city 22

SI.1 General crime trends in Nottingham city from 2012 to 2019

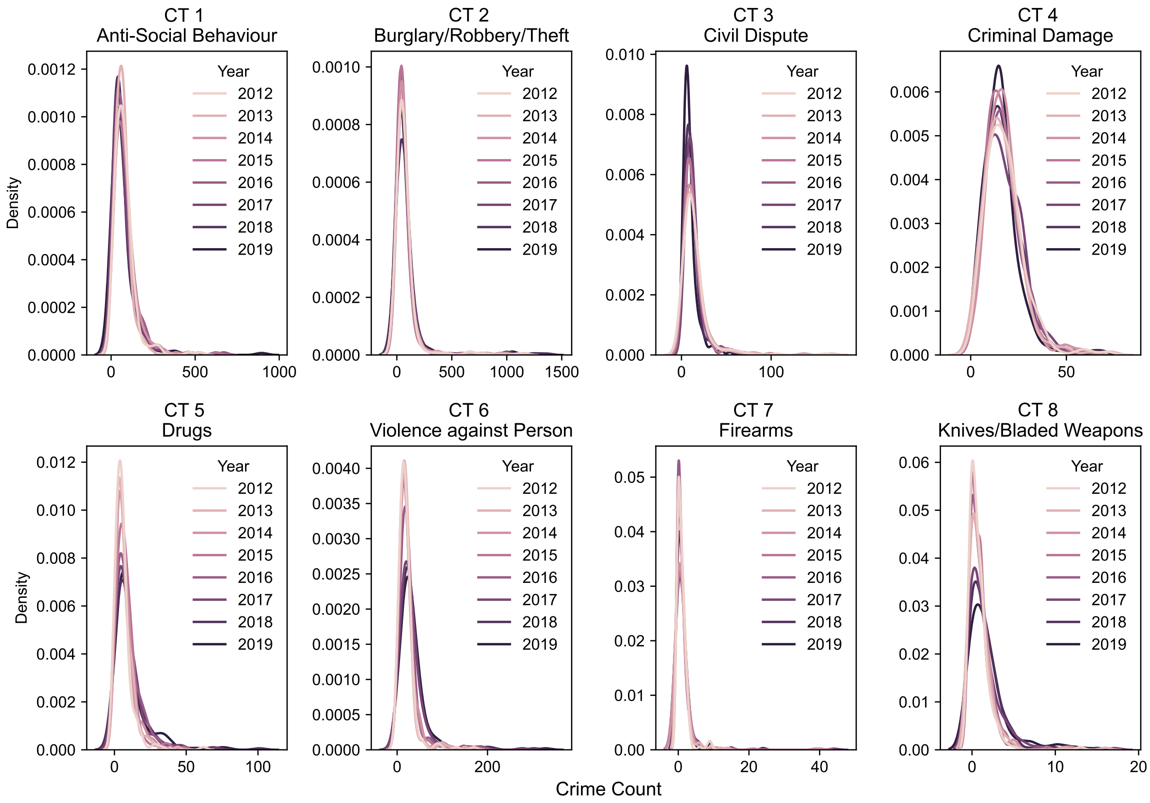

Figure S1: **Density histograms of 8 crime types across 182 LSOAs between 2012 and 2019.** While the counts of the 8 crimes varied in different years, the shapes of the distribution plots of the 8 crime types do not change much (i.e., no extremely skewed distribution), which shows the overall trend under each crime is relatively stable over the 8 years.

Table S1: **Average annual crime counts of eight crime types across twenty wards in Nottingham between 2012 and 2019**

**Ward**	$\overline{\textbf{CT1}}$ **(Anti-Social Behaviour)**	$\overline{\textbf{CT2}}$ **(Burglary/ Robbery/ Theft)**	$\overline{\textbf{CT3}}$ **(Civil Dispute)**	$\overline{\textbf{CT4}}$ **(Criminal Damage)**	$\overline{\textbf{CT5}}$ **(Drugs)**	$\overline{\textbf{CT6}}$ **(Violence against Person)**	$\overline{\textbf{CT7}}$ **(Firearms)**	$\overline{\textbf{CT8}}$ **(Knives/ Bladed Weapons)**
Arboretum	1134.8	856.8	176.1	169.4	125.4	357.8	9.1	15.8
Aspley	708.3	494.4	120.6	217.0	70.5	273.5	8.6	10.1
Basford	543.6	518.6	111.9	162.5	59.9	219.0	14.9	7.8
Berridge	951.3	766.1	176.4	188.1	98.3	262.8	7.5	14.8
Bestwood	699.1	361.1	87.5	222.0	71.1	219.0	6.0	9.0
Bilborough	701.0	446.1	102.5	180.8	61.6	232.4	13.4	8.6
Bridge	1923.1	2426.8	259.5	254.4	208.6	627.4	12.1	30.5
Bulwell	828.6	808.8	135.8	247.8	75.0	281.0	8.9	11.3
Bulwell Forest	372.0	388.4	65.1	132.5	45.1	165.4	3.9	6.3
Clifton North	273.6	294.1	54.1	102.4	38.9	115.5	5.1	5.3
Clifton South	487.5	399.5	71.8	132.5	55.0	172.6	7.3	7.5
Dales	731.8	446.9	128.4	161.1	84.1	230.3	10.5	10.1
Dunkirk and Lenton	406.1	537.0	44.3	75.3	39.6	147.5	18.4	10.6
Leen Valley	301.0	369.5	91.9	86.1	36.1	128.6	20.6	4.5
Mapperley	507.4	443.8	101.3	121.8	67.4	185.3	8.8	7.9
Radford and Park	924.5	924.5	180.3	155.9	88.8	243.8	9.8	12.4
Sherwood	424.9	594.3	91.0	134.1	60.6	238.3	5.3	10.4
St Ann's	1421.5	1775.5	290.3	240.3	179.0	522.3	18.0	24.4
Wollaton East and Lenton Abbey	130.0	271.3	29.6	38.6	19.5	43.1	2.4	1.4
Wollaton West	164.8	376.8	44.6	54.5	24.1	52.6	2.8	3.1
**Total**	13634.8	13500.0	2362.8	3076.9	1508.6	4717.9	193.1	211.5

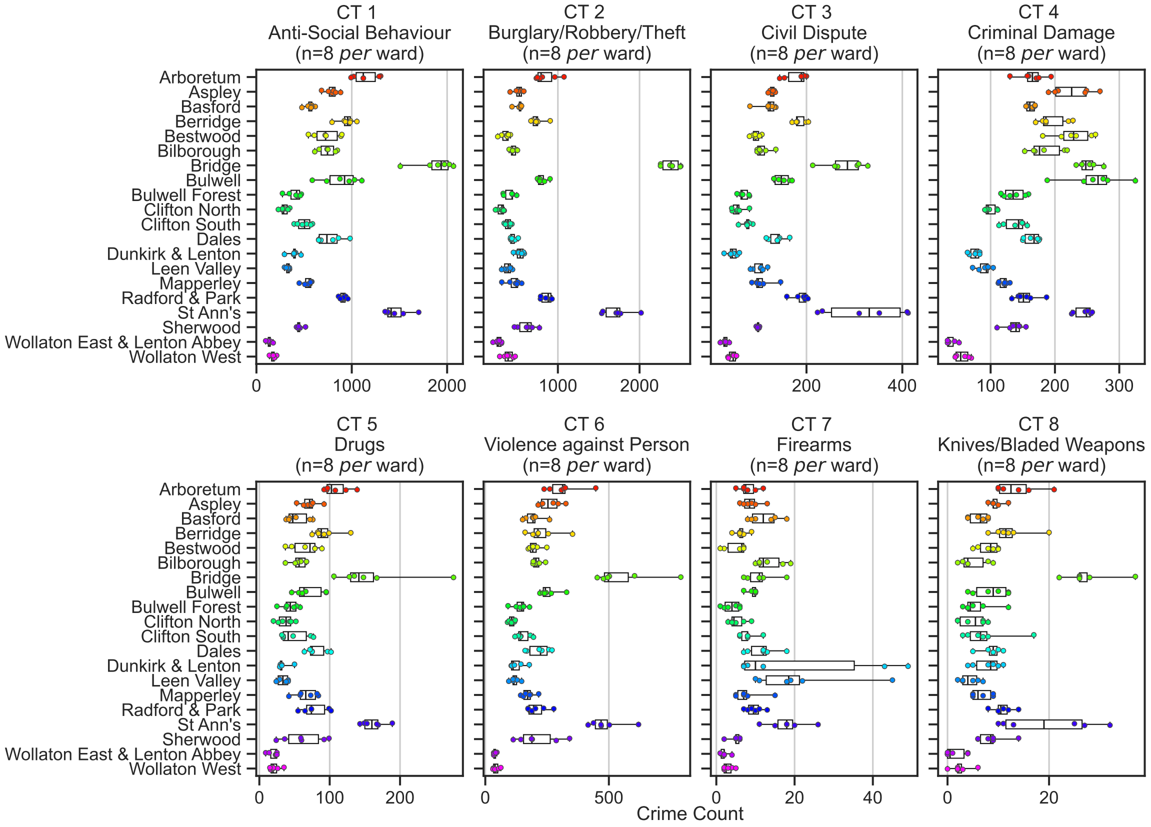

Figure S2: **Boxplot of eight crime types (CTs) in twenty wards in Nottingham between 2012 and 2019.** Each dot denotes one year's data on the related CT and ward. Apart from Bridge and St Ann's, the rest of the wards in the city of Nottingham usually had relatively stable crime counts under 8 CTs in 2012-2019, as the lengths of boxes of these two wards in most of the 8 CTs are larger than the others. Boxplots present the 25th, 50th and 75th percentiles, and whiskers represent the full range of the values.

SI.2 Selected socioeconomic features

Table S2: **Description of selected socioeconomic features**

Feature	Description	Source
Population density	Number of persons per hectare.	Census for England and Wales (2011)
Population aged 15-24 years (%)	Proportion of the total population aged between 15 and 24.	Census for England and Wales (2011)
Highest level of qualification: level 4 qualifications and above	Refers to degree (BA, BSc), higher degree (MA, PhD, PGCE), NVQ level 4 to 5, HNC, HND, RSA Higher Diploma, BTEC Higher level, professional qualifications (for example, teaching, nursing, accountancy).	Census for England and Wales (2011)
Born outside the UK (%)	Proportion of the resident population not born in the UK.	Census for England and Wales (2011)
Social rented (%)	Proportion of the total population rents from council or local authority.	Census for England and Wales (2011)
Income deprivation domain	Proportion of the total population in an area experiencing deprivation relating to low income. Higher score means higher deprivation.	Index of Multiple Deprivation, Office for National Statistics (2015)
Employment deprivation domain	Proportion of the working-age population in an area involuntarily excluded from the labour market. Higher score means higher deprivation.	Index of Multiple Deprivation, Office for National Statistics (2015)
Health deprivation and disability domain	Risk of premature death and the impairment of quality of life through poor physical or mental health. Higher score means higher deprivation.	Index of Multiple Deprivation, Office for National Statistics (2015)
Barriers to housing and services domain	Physical and financial accessibility of housing and local services. The indicator measures both physical proximity of local services and issues relating to access to housing such as affordability and homelessness.	Index of Multiple Deprivation, Office for National Statistics (2015)

Note: Features collected from Census for England and Wales (2011) [1] and Index of Multiple Deprivation, Office for National Statistics (2015) [2] were produced at LSOA level and were aggregated to the ward level by summing the population-weighted scores of the corresponding LSOAs in the ward [3].

SI.3 Field interviews

We conducted 13 interviews with police officers in Nottingham from 2022 to 2023. We obtained consent from all interviewees before we started the interviews. Table S3 shows the brief information of these field interviews.

Table S3: **Basic information of field interviews**

Interviewee	Position	Area of Patrol	Interview Date
Officer 1	Police Officer, Nottingham	City South (The Meadows and Sneinton)	29.06.2022
Officer 2	Police Officer, Nottingham	City South	29.06.2022
Officer 3	Police Officer, Nottingham	City South (St Ann’s, Sneinton and Meadows)	29.06.2022
Officer 4	Police Officer, Nottingham	City Centre	29.06.2022
Officer 5	Police Officer, Nottingham	City Centre	29.06.2022
Officer 6	Police Officer, Nottingham	City North	08.08.2022
Officer 7	Police Officer, Nottingham	City South (St Ann’s, Meadows)	08.08.2022
Officer 8	Police Officer, Nottingham	City Centre (Radford)	08.08.2022
Officer 9	Police Officer, Nottingham	City Centre	08.08.2022
Officer 10	Police Officer, Nottingham	Bestwood	01.03.2023
Officer 11	Police Officer, Nottingham	Bestwood	31.03.2023
Officer 12	Police Officer, Nottingham	City Centre (St Ann’s, Sneinton)	09.05.2023
Officer 13	Police Officer, Nottingham	Chief Inspector in charge of City North, South and Central West	09.05.2023

Below are the key points we obtained from the interviews:

- “In Bestwood he was God-like” told us officer 2. When Colin’s nephew died, he expected residents to put obituaries in the local paper to the point that the paper had to hire extra staff. There was a horse drawn cart that went through the middle of Bestwood, with a procession of gangsters – officer 10
- *[policing theft]* An officer who had worked in the estate for more than twenty years in the nineties and early 2000s recalls that, in his time on the estate, “you didn’t commit burglaries or car thefts or the like on the Bestwood Estate because Colin would police that himself and he was more effective in dealing with it than the police.” For instance, “a local teacher got her car broken into and, by the time the police get there, it’s been resolved by Colin, ‘no I’ve got my property. Colin sorted that out I don’t need you,’ she told me.” (officer 10). His house was “almost like a castle on the hill, in the middle of the estate”, for all to see (officer 10) and he cultivated an imagine of a local Robin Hood. “I think he probably enjoyed that [role] more than supplying drugs, he loved being that local governance within the community, the person that people looked to and feared” (officer 10; similarly, see also [4]).

  “the crimes that got reported in Bestwood tend to be firearms or assault or criminal damage” – officer 10

  “These crimes are an indicator of Colin putting somebody back into their place, back in line” – officer 10

  When somebody broke into the shed of a relative of Colin we were on high alert, because something serious could happen to the thief. – officer 10

  Colin lent money – officer 10

Colin would position people watching cars coming in and out of the Bestwood police car park – office 10

Colin was born and grew up in Bestwood – officer 10

A lot of people in the community are his relative and friends – officer 10

"robin hood type" – officer 10

Colin had direct contacts with gangsters and drug suppliers in Manchester and Liverpool – officer 10

Control of territory – officers 10, 11

Paid for firework display raffle prizes for local schools at Christmas – officer 10

Lack of trust in police within the community – officer 10, 11

- *[generosity]* Colin and his peers were capable of acts of generosity towards members of the community who were struggling financially and paid for firework display and raffle prizes for the local schools (officer 10; [4]: 115). In his return for his generosity, Colin demanded loyalty, asked for favours and his victims could not avoid several punishments if they refused. [the reputation lingers on: "you still get people saying 'well, I'm—you know–, I am Colin Gunn's second cousins forth removed former roommate' – officer 2

Officer 3: Bestwood cartel is still active.

Officer 8: "there have been a couple of instances of a couple of people that I have dealt with where they have said 'I know Colin'" to increase their standing.

"he will not let his influence on the neighbourhood go" – officer 10

Colin still manages to community from prison through hidden cell phones, or in coded conversation on the official phone – officer 10 (see also [5])

Even today Colin's name is used when two people are in a dispute or a fight. But Colin and his gang would charge if somebody used their name. He would give people authority to use his name. – officer 10

Colin does not come from a criminal family – officer 10

Surely there has been a decline in his power. – officer 10

Criminal damage and damage to property is a warning sent coming from the OCG to a person who is out of line. Criminal damage includes arson attacks in the data. – officer 10

Although arson is not very common. It would be more common smashing a window or damaging a car, and it is a form of intimidation – officer 10

Reach in every part of town – officer 11

People in the neighbourhood would not notice that there was a powerful OCG in Bestwood. People think that they are running in a war zone, but "good", organised crime don't operate like that. They stay under the radar. They are quite orderly. – officer 11

Still in my time if people tried to enter the territory, Colin's people would come and stop them – officer 11

Many of the pubs closed, so it reduced high power! – officer 11

His influence is surely diminished but he still communicates from prison so he can still exercise influence – officer 11

When in 2020 Colin's niece was slapped in public, in 24 hours the person who did was punished/shot in the leg. Sign of continuing influence – officer 11

His Porsche was a display of power – officer 11

His name carried a lot of power – officer 11

It was not that they trusted him a lot. There was a lack of trust in us. – officer 11

He absolutely still has got influence – officer 11

- *[Other parts of Nottingham]* Officers patrolling the City Centre and the South of the city report high level of crime, but no community engagement of the kind seen in Bestwood or attempts to corrupt public officials, reduce anti-social behaviour, settle business disputes, or run protection rackets (officer 1, officer 2). "There is no Robin Hood," officer 1 told us. In neighbourhoods such as St Ann's and The Meadows (Bridge) they observe 'urban street gangs', dealing in drugs and robberies, with no ability to engage with the community or corruption officers and officials. "They stab and beat up each other's" said officer 2 (see also officer 1). Prostitution is linked to consumption of illegal substance. Corrupting police officers and local officials is "an echelon too high for them" (officer 2). Officers also note that in community meetings local urban gangs are not discussed as a major concern, suggesting that their impact is anyway low (e.g. officer 1). Often such groups do not even have a name (officer 2).

  These gangs do not last long – officer 3 "what we are seeing in Sneinton is 15-17 years old kids running around with machetes, get knocked off push cycles with 60 gran of class A drugs in their pocket" – officer 3

  Gangs in Central and south exist to control the drugs market – officer 3

  City center they commit lots of shoplifting – officer 3

  No community activities in south and central – officer 3

  They are not organised – officer 3

  No protection rackets – officer 3

  They commit spontaneous robberies – officer 3

  These gangs are held in contempt by community – officer 3

  No protection rackets no interaction with the community in city center – officer 4

  No corruption no protection racket city center – officer 5

  Bulwell, there is one OCG there. They are just the new fashion ones, and they just concentrate on their drug lines and make sure they keep their drug lines. They don't intervene in solving disputes, attempting to control antisocial behaviour, or running a protection racket within the community. They are just dealers, and they just fall out with each other, and it's self-contained – officer 6

  St Ann's, OCG is quite prevalent, but they are urban street gangs. They do drugs and violent crimes nothing else – officer 7

  City central. Also nothing like protection rackets corruption or influence in the community – officer 8

  City central. Many students. Also nothing like protection rackets corruption or influence in the community – officer 9

  In Nottingham there are some ethnic crime groups. Kurds who do cigarettes and drugs. They are a kind of mafia. Same for Roma gypsies. They have their own roma courts etc. they are in Radford road – officer 9

  In Forest fields there are Somali and polish communities – officer 9

SI.4 Crimes in Bestwood and Bulwell

Utilising the observed yearly crime counts from Bestwood and Bulwell from 2012 to 2019, we conducted Welch's t-test to check if Bestwood is significantly different from its matched pair Bulwell on individual crime type. Furthermore, we bootstrap the observed values of Bestwood and Bulwell 10,000 times for each CT, respectively, to obtain a distribution of the 10,000 Welch's t-statistics for individual CT, offering a more robust statistical overview than the single-shot observed test.

In Fig. S3, we can see that Bestwood is significantly lower than its matched pair Bulwell on every CT. The quantitative analyses resonate with our qualitative research, that is, the presence of Colin's group in Bestwood reduced certain crimes.

We also use the other measurement, crime count, to do the Welch's t-test. Even though not considering the effect of population density, Bestwood is still significantly lower than Bulwell on Burglary/Robbery/Theft (CT 2), Civil Dispute (CT 3), Violence against Person (CT 6), and Firearms (CT 7), As shown in Fig. S4 and S5.

Table S4: **Matching similarities of the rest of Nottingham wards to Bestwood**

**Ward**	**KNN-Manhattan Distance**		**KNN-Euclidean Distance**	
	Similarity Ranking (from high to low)	Value	Similarity Ranking (from high to low)	Value
Bulwell	1	0.9387	1	0.4005
Bilborough	2	0.9752	2	0.4207
Aspley	3	1.0661	3	0.4325
Clifton South	4	1.1238	4	0.4418
Basford	5	1.1320	5	0.4454
Dales	6	1.5113	6	0.6178
St Ann's	7	1.8221	7	0.7715
Bulwell Forest	8	2.0262	8	0.8102
Clifton North	9	2.1028	9	0.8441
Mapperley	10	2.1789	11	0.8689
Sherwood	11	2.1902	12	0.8759
Leen Valley	12	2.2038	10	0.8563
Arboretum	13	2.2883	13	0.9103
Berridge	14	2.4438	15	0.9819
Bridge	15	2.5501	14	0.9679
Radford and Park	16	3.6581	16	1.2714
Wollaton East and Lenton Abbey	17	3.8723	17	1.3795
Dunkirk and Lenton	18	4.0559	18	1.4338
Wollaton West	19	4.0716	19	1.6005

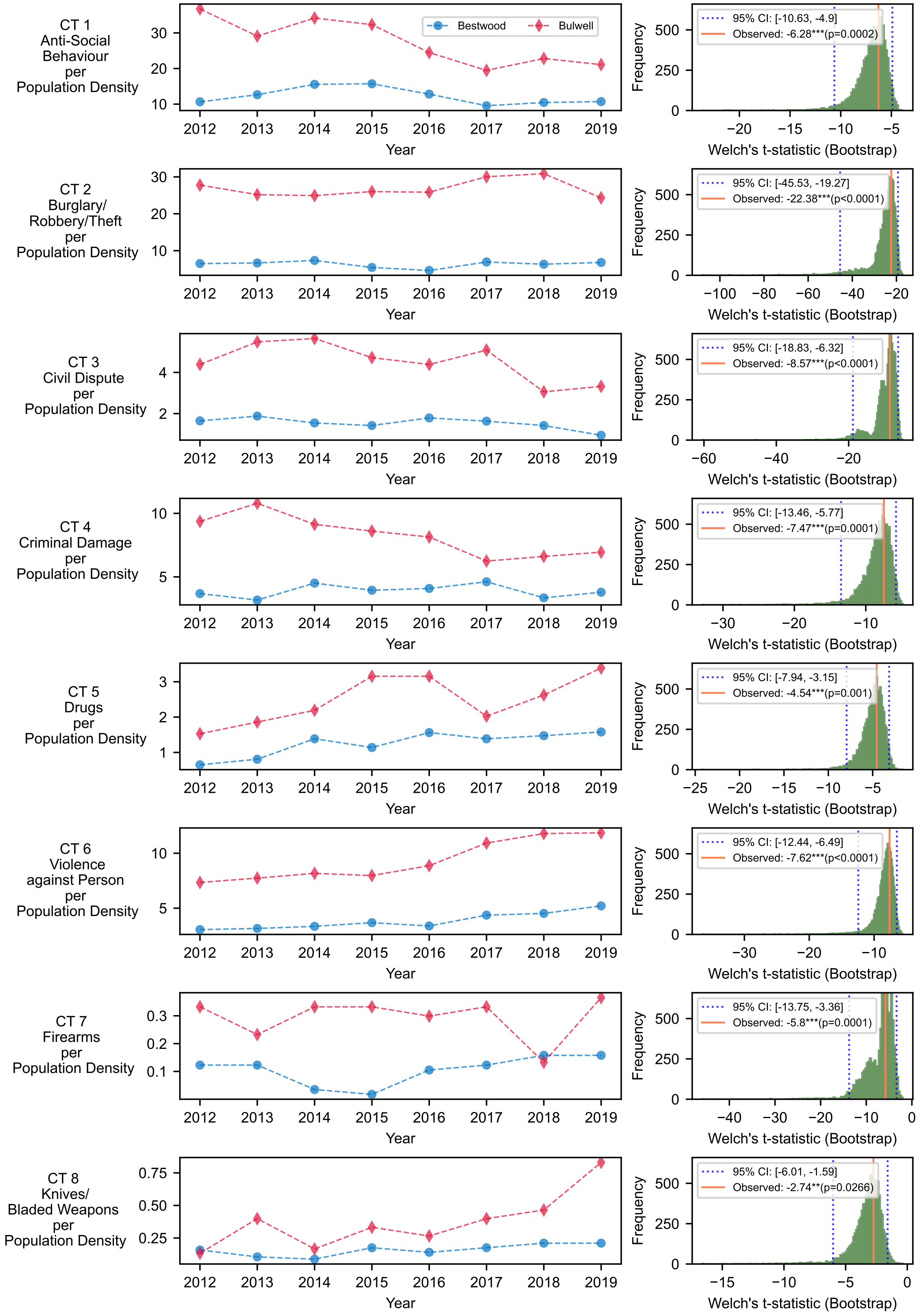

Figure S3: **Yearly crime counts per population density of eight CTs in Bestwood and Bulwell (2012–2019) and their statistical differences.** The left panel shows the observed crime counts per population density of each CT from 2012 to 2019. The right panel shows the histograms of two-sided Welch's t-tests of the associated left, wherein we further bootstrap the left observed values 10,000 times of each CT, respectively. In general, Bestwood is significantly lower than Bulwell regarding all eight crime measurements. The 95% Confidence Interval (CI) of the bootstrapped Welch's t-test distributions further indicate the differences are significant. $^{***}p < 0.01$, $^{**}p < 0.05$, $^{*}p < 0.1$.

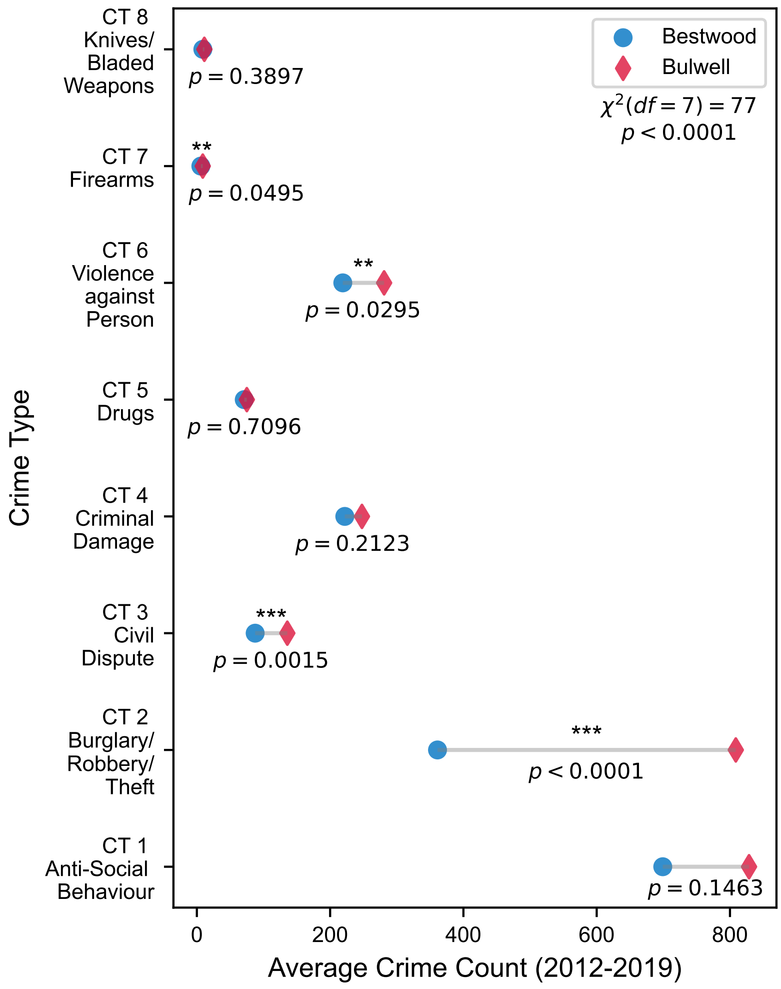

Figure S4: **Matching comparison between Bestwood and Bulwell on eight crime types measuring by average crime counts.** Overall, Bulwell displays higher average crime counts than Bestwood across all eight crime types (CTs), though the differences are minimal for Drugs (CT 5), Firearms (CT 7), and Knives/Bladed Weapons (CT 8). The χ^2 test (two-sided), based on average crime counts, reveals that the two matched wards differ significantly ($p < 0.0001$) with respect to the eight CTs. The grey bars illustrate the differences in crime rates for each CT between the two wards. The p values under the bars for Burglary/Robbery/Theft (CT 2), Civil Dispute (CT 3), Violence against Person (CT 6), and Firearms (CT 7) indicate that Bestwood's crime counts are significantly lower than those of Bulwell from 2012 to 2019, as determined by two-sided Welch's t-tests (see Fig. S5). $^{***}p < 0.01$, $^{**}p < 0.05$, $^{*}p < 0.1$.

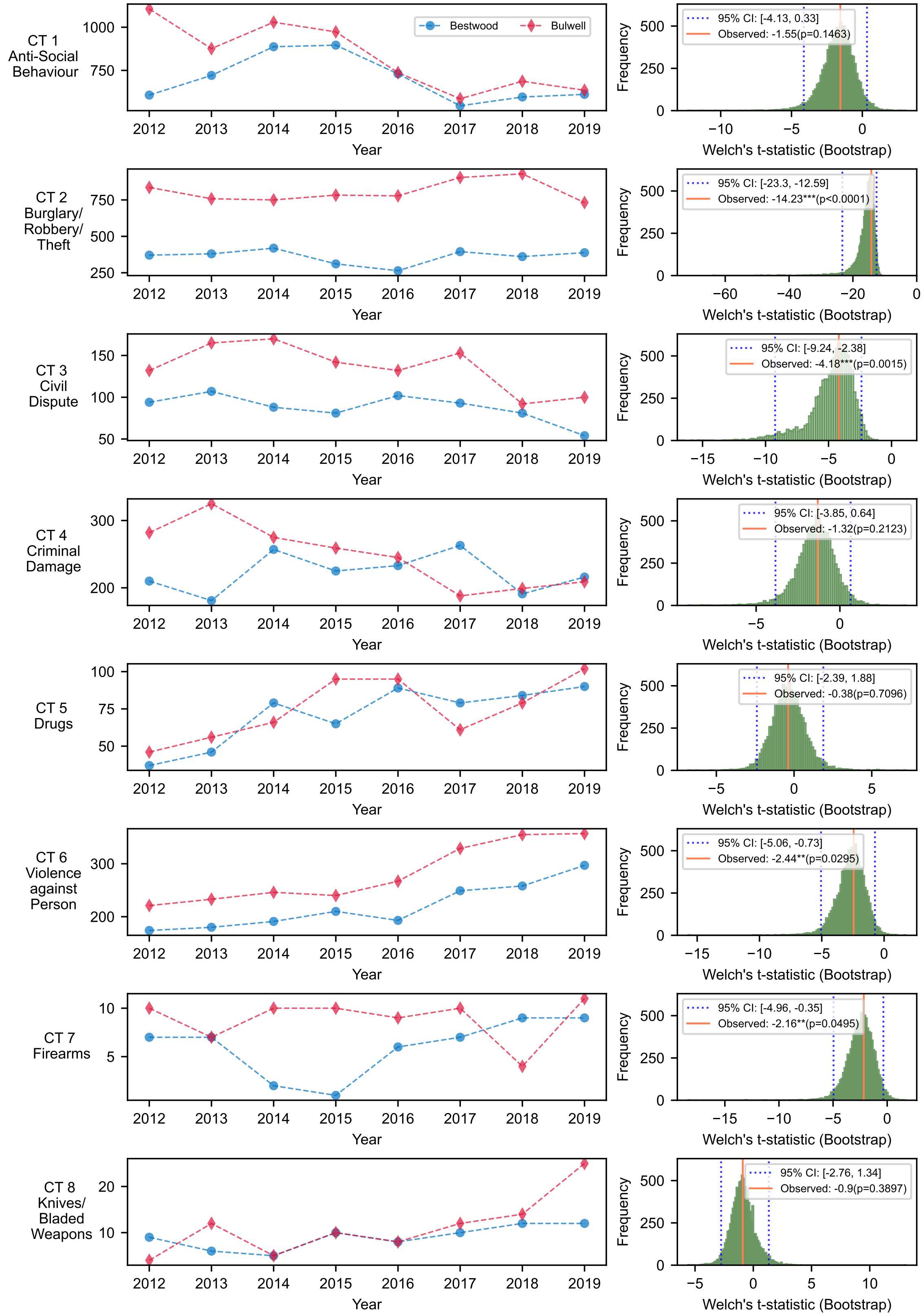

Figure S5: **Yearly crime counts of eight crime types in Bestwood and Bulwell (2012—2019) and their statistical differences.** The left panel shows the observed crime counts of each CT from 2012 to 2019. The right panel shows the histograms of Welch's t-test (two-sided) of the associated left, wherein we further bootstrap the left observed crime counts 10,000 times of each CT, respectively. The 95% Confidence Interval (CI) of the bootstrapped Welch's t-test distributions indicate the statistical significance again, e.g., the difference is insignificant when the statistic 0 sits in the 95% CI. $^{***}p < 0.01$, $^{**}p < 0.05$, $^{*}p < 0.1$.

SI.5 Crimes in Bestwood and its second-most similar and nearby wards

In addition to the matched pair Bulwell, we have also investigated the difference between Bestwood with its second-most similar ward, Bilborough, as well as its three nearby wards, i.e., Basford, Bulwell Forest, and Sherwood, under the eight CTs. The comparisons were conducted using yearly crime count per population density and yearly crime count measurements. As Table S4 shows, Bilborough is the second-most similar ward to Bulwell, which can be a logic robustness check. Additionally, the three neighbouring wards of Bestwood are not very similar to Bestwood in terms of socioeconomic features. Notably, Bestwood's population density is the highest among the four wards while close to Sherwood, according to Table 1.

According to Fig. S6, Bestwood is significantly lower than Bilborough on every crime type other than CT 8, when considering the population density effect. Fig. S7 shows that Bestwood is still significantly lower than Bilborough on CT 2, CT 4, and CT 7, when measured by population.

For the difference between Bestwood and Basford, Fig. S8 shows that Bestwood is significantly lower than Basford on CT 2, CT 3, CT 6, and CT 7, when considering the population density effect. Fig. S9 shows that Bestwood is significantly lower than Basford on CT 2, CT 3, and CT 7. Meanwhile, Bestwood is significantly higher than Basford on CT 1 and CT 4.

Comparing with Bulwell Forest, Bestwood is still significantly lower than Bulwell Forest on CT 2 when measuring on crime count per population density, as indicated in Fig. S10. Meanwhile, Bestwood is only significantly higher than Bulwell Forest on CT 1 and CT 4. When measuring on crime count, Bestwood is significantly higher on several crimes, such as CT 1, CT 3, CT 4, CT 5, CT 6, and CT 7, as Fig. S11 shows.

Moving to Sherwood, Bestwood is significantly lower than Sherwood on CT 2 and CT 3, although it is significantly higher than Sherwood on CT 1 and CT 4 (see Fig. S12), when using the crime count per population density measurement. Regarding the crime count measurement, Bestwood is still significantly lower than Sherwood on CT 2, as Fig. S13 shows. Meanwhile, Bestwood is significantly higher than Sherwood on CT 1 and CT 4.

Overall, in most of the cases, Bestwood is significantly lower than its three neighbours on CT 2 (Burglary/Robbery/Theft); many times, we can also see Bestwood is lower than its neighbours on CT 3 (Civil Dispute), which provide statistical evidence for supporting the interviews. Besides, Bestwood is also significantly higher than its neighbours on a few other crimes when measuring on crime count, such as CT 1 (Anti-Social Behaviour) and CT 4 (Criminal Damage), which indicates Colin's group still commits certain crimes in the community. However, we again point out that the three neighbours are not very similar to Bestwood in terms of socioeconomic features when one digests the comparisons. Nevertheless, these results supply extra insights into the matching comparison between Bestwood and Bulwell.

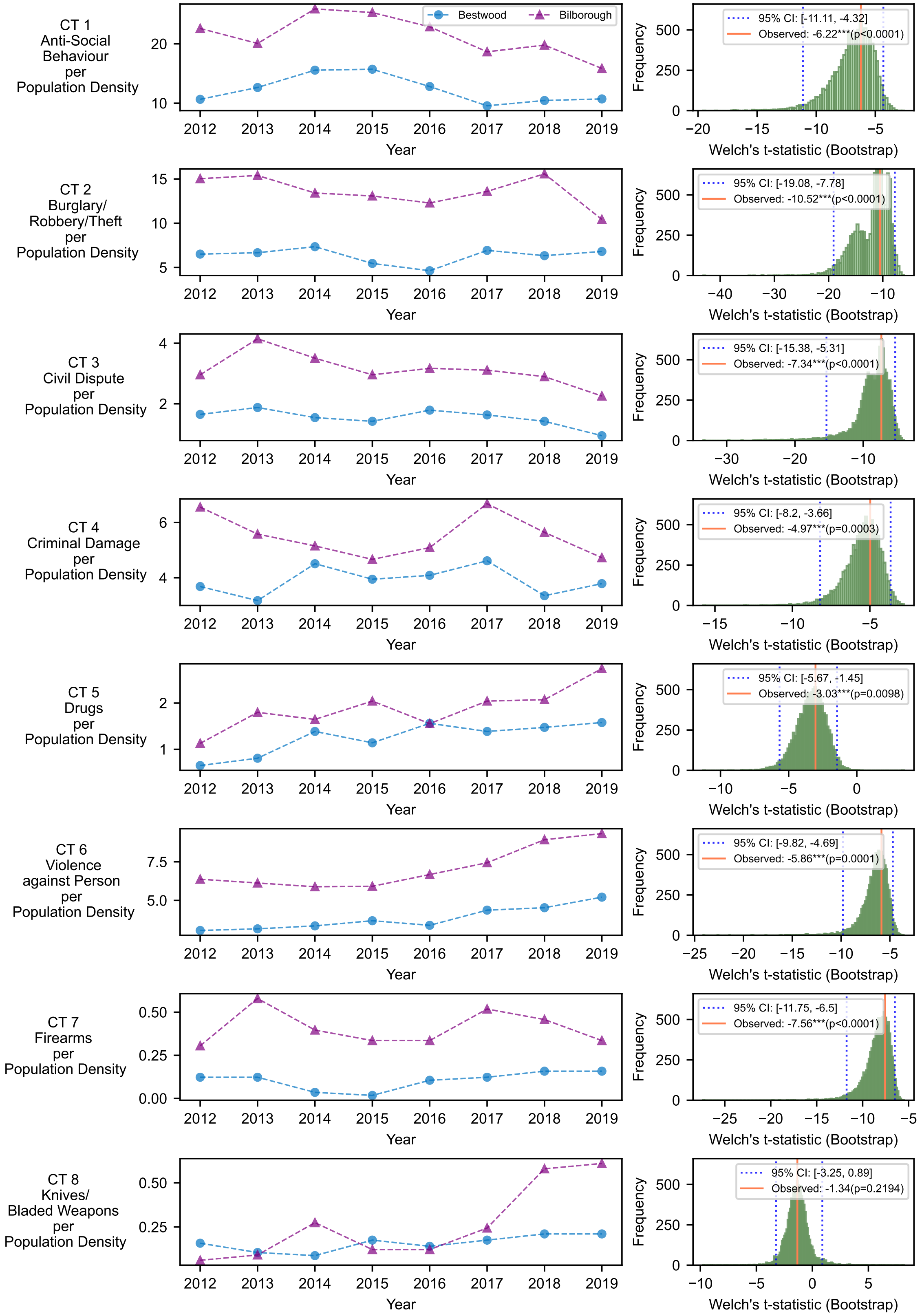

Figure S6: **Yearly crime counts per population density of eight crime types in Bestwood and Bilborough (2012—2019) and their statistical differences.** The left panel shows the observed measurements of Bestwood and Basford under eight CTs, respectively. The right panel shows the associated Welch's t-statistics (two-sided) from both the observed values and 10,000 bootstrapped values. $^{***}p < 0.01$, $^{**}p < 0.05$, $^{*}p < 0.1$.

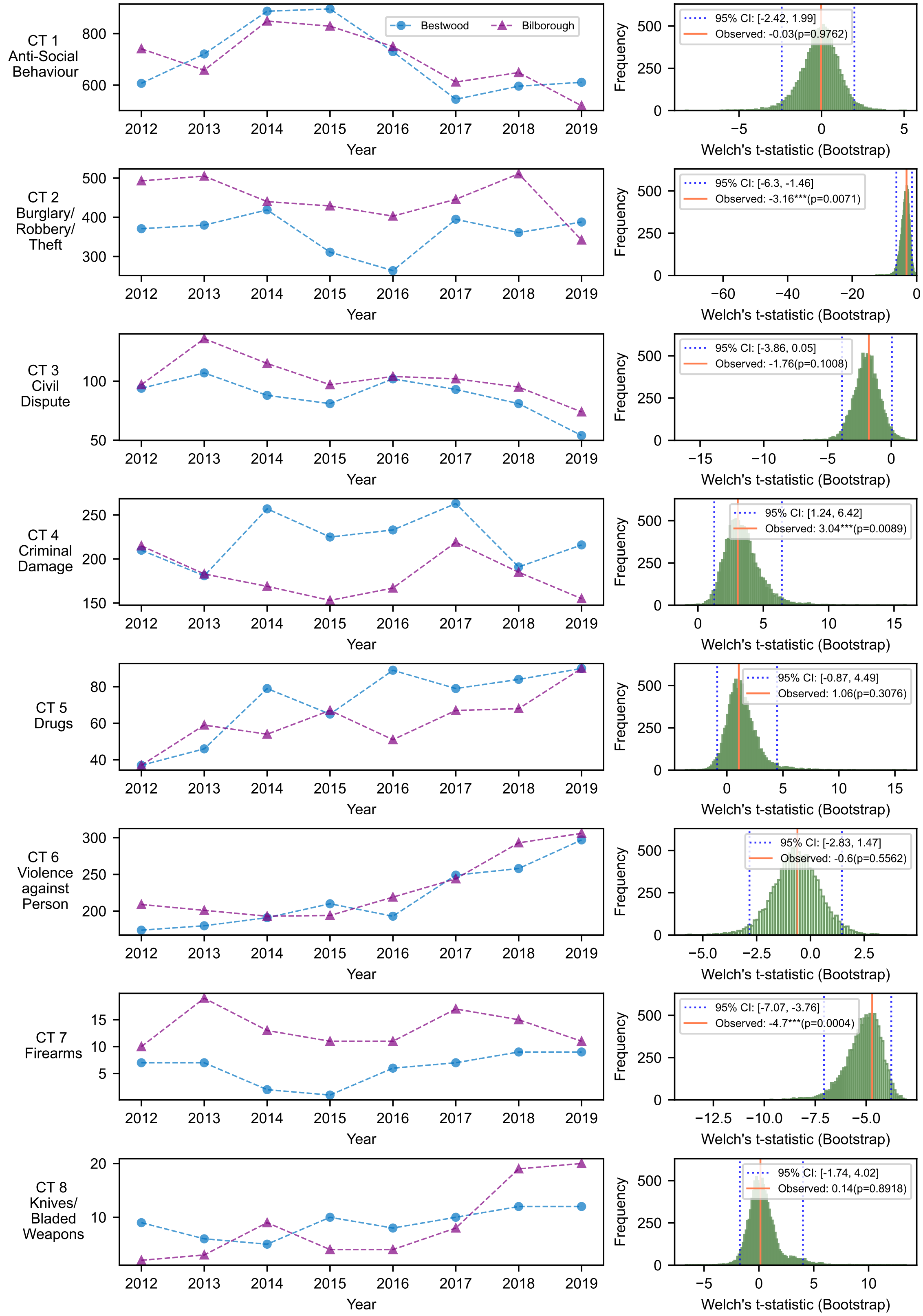

Figure S7: **Yearly crime counts of eight crime types in Bestwood and Bilborough (2012—2019) and their statistical differences.** The left panel shows the observed measurements of Bestwood and Basford under eight CTs, respectively. The right panel shows the associated Welch's t-statistics (two-sided) from both the observed values and 10,000 bootstrapped values. $^{***}p < 0.01$, $^{**}p < 0.05$, $^{*}p < 0.1$.

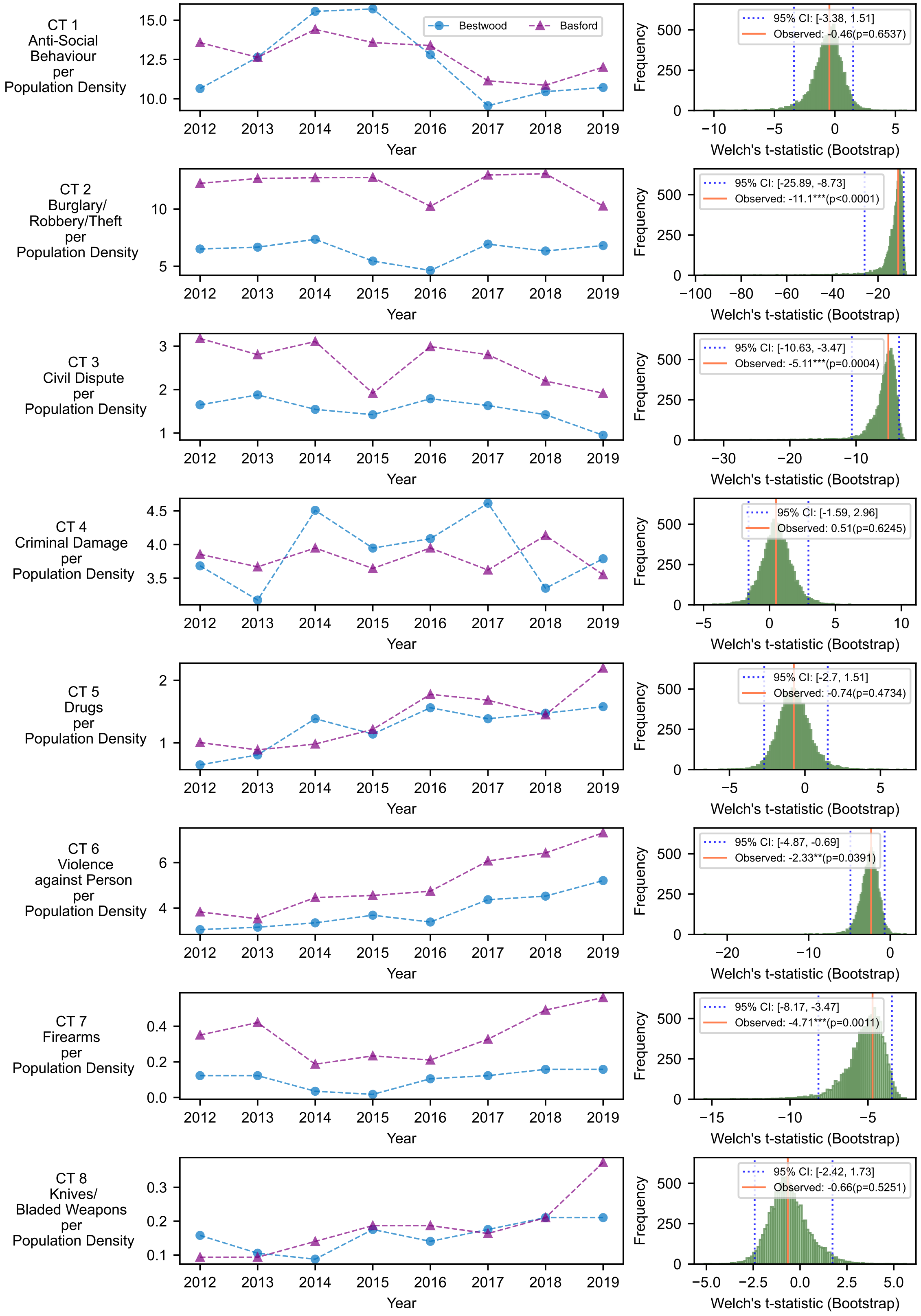

Figure S8: **Yearly crime counts per population density of eight crime types in Bestwood and Basford (2012–2019) and their statistical differences.** The left panel shows the observed measurements of Bestwood and Basford under eight CTs, respectively. The right panel shows the associated Welch's t-statistics (two-sided) from both the observed values and 10,000 bootstrapped values. $^{***}p < 0.01$, $^{**}p < 0.05$, $^{*}p < 0.1$.

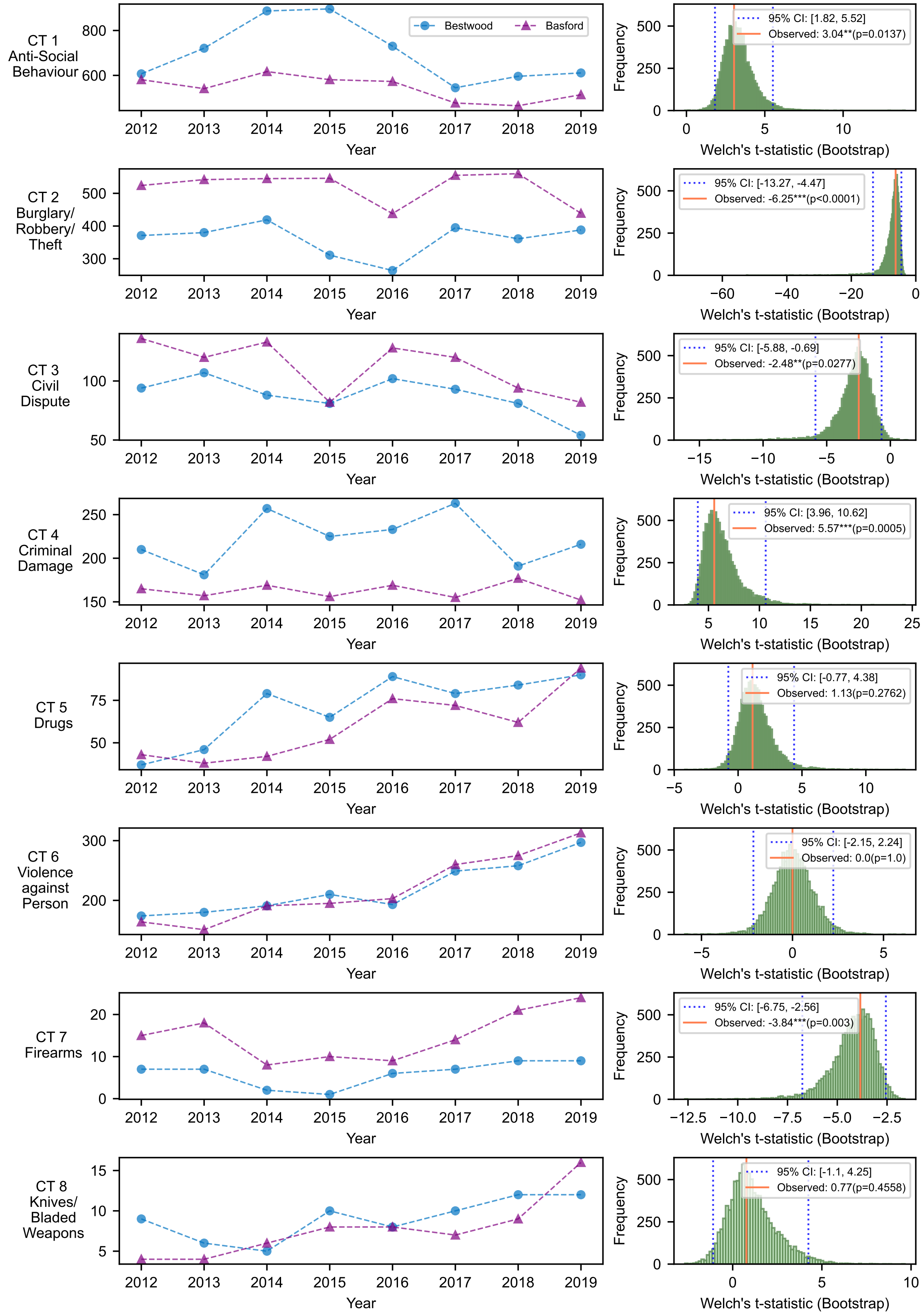

Figure S9: **Yearly crime counts of eight crime types in Bestwood and Basford (2012—2019) and their statistical differences.** The left panel shows the observed measurements of Bestwood and Basford under eight CTs, respectively. The right panel shows the associated Welch's t-statistics (two-sided) from both the observed values and 10,000 bootstrapped values. $^{***}p < 0.01$, $^{**}p < 0.05$, $^{*}p < 0.1$.

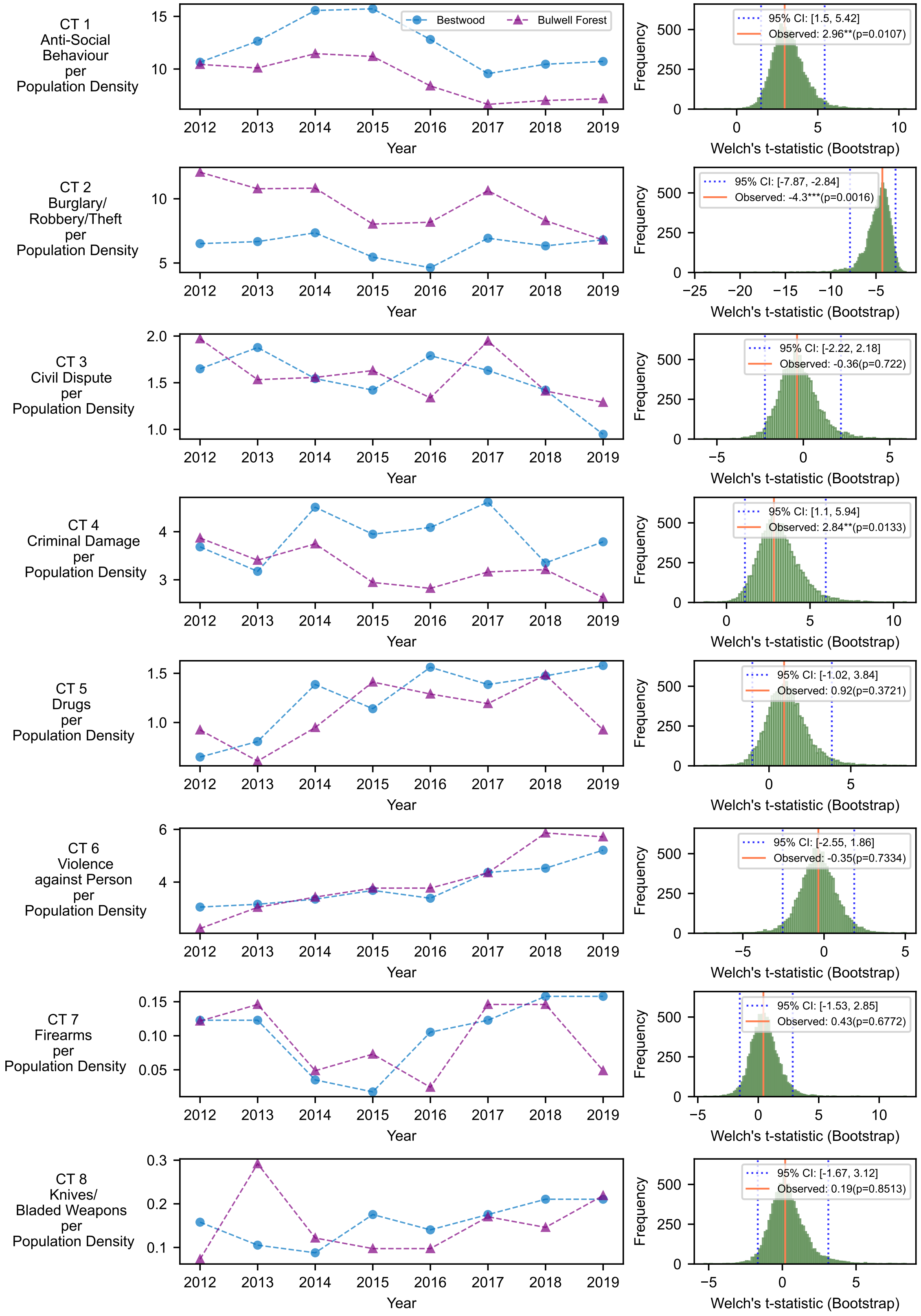

Figure S10: **Yearly crime counts per population density of eight crime types in Bestwood and Bulwell Forest (2012—2019) and their statistical differences.** The left panel shows the observed measurements of Bestwood and Bulwell Forest under eight CTs, respectively. The right panel shows the associated Welch's t-statistics (two-sided) from both the observed values and 10,000 bootstrapped values. $^{***}p < 0.01$, $^{**}p < 0.05$, $^{*}p < 0.1$.

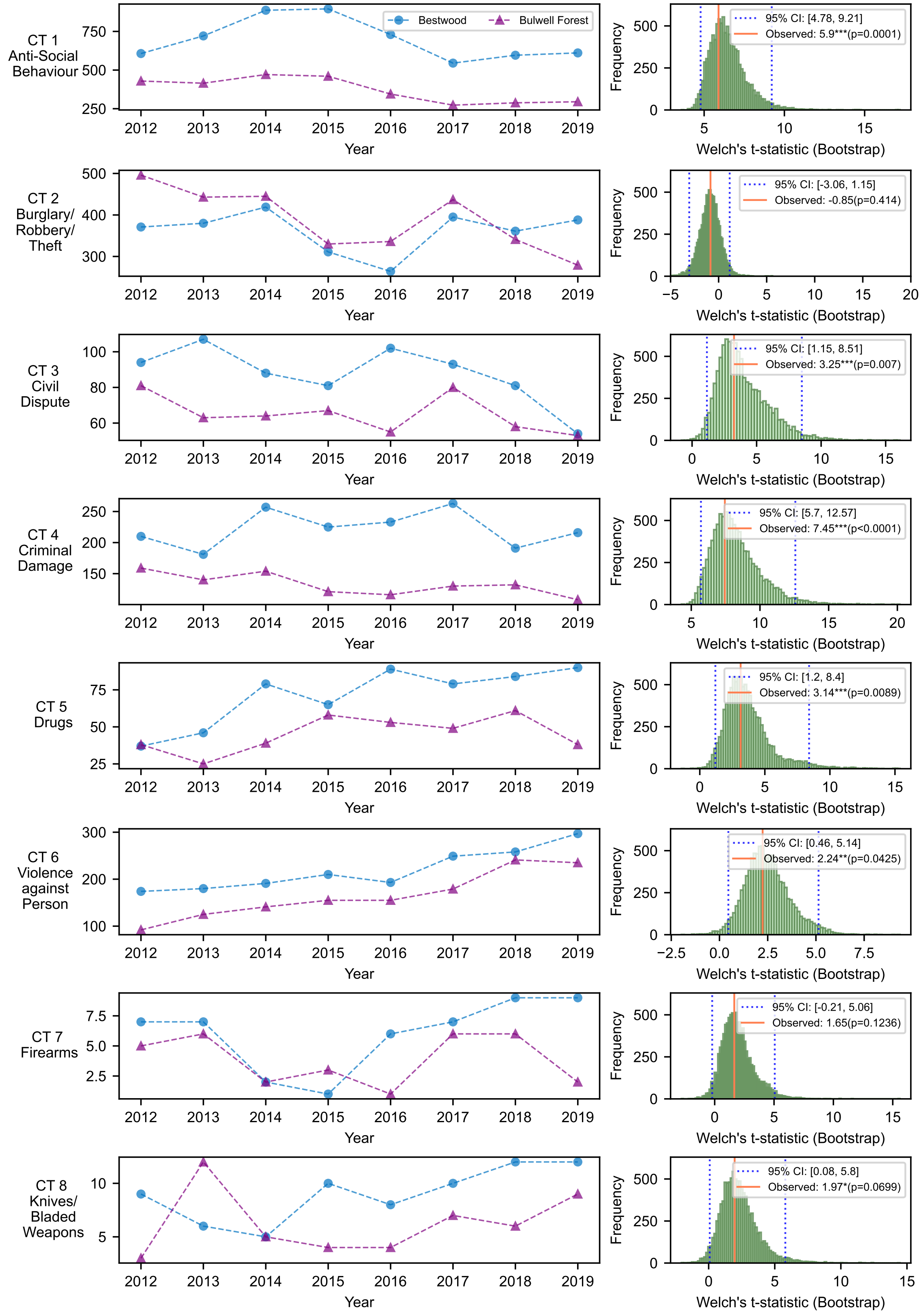

Figure S11: **Yearly crime counts of eight crime types in Bestwood and Bulwell Forest (2012—2019) and their statistical differences.** The left panel shows the observed measurements of Bestwood and Bulwell Forest under eight CTs, respectively. The right panel shows the associated Welch's t-statistics (two-sided) from both the observed values and 10,000 bootstrapped values. $^{***}p < 0.01$, $^{**}p < 0.05$, $^{*}p < 0.1$.

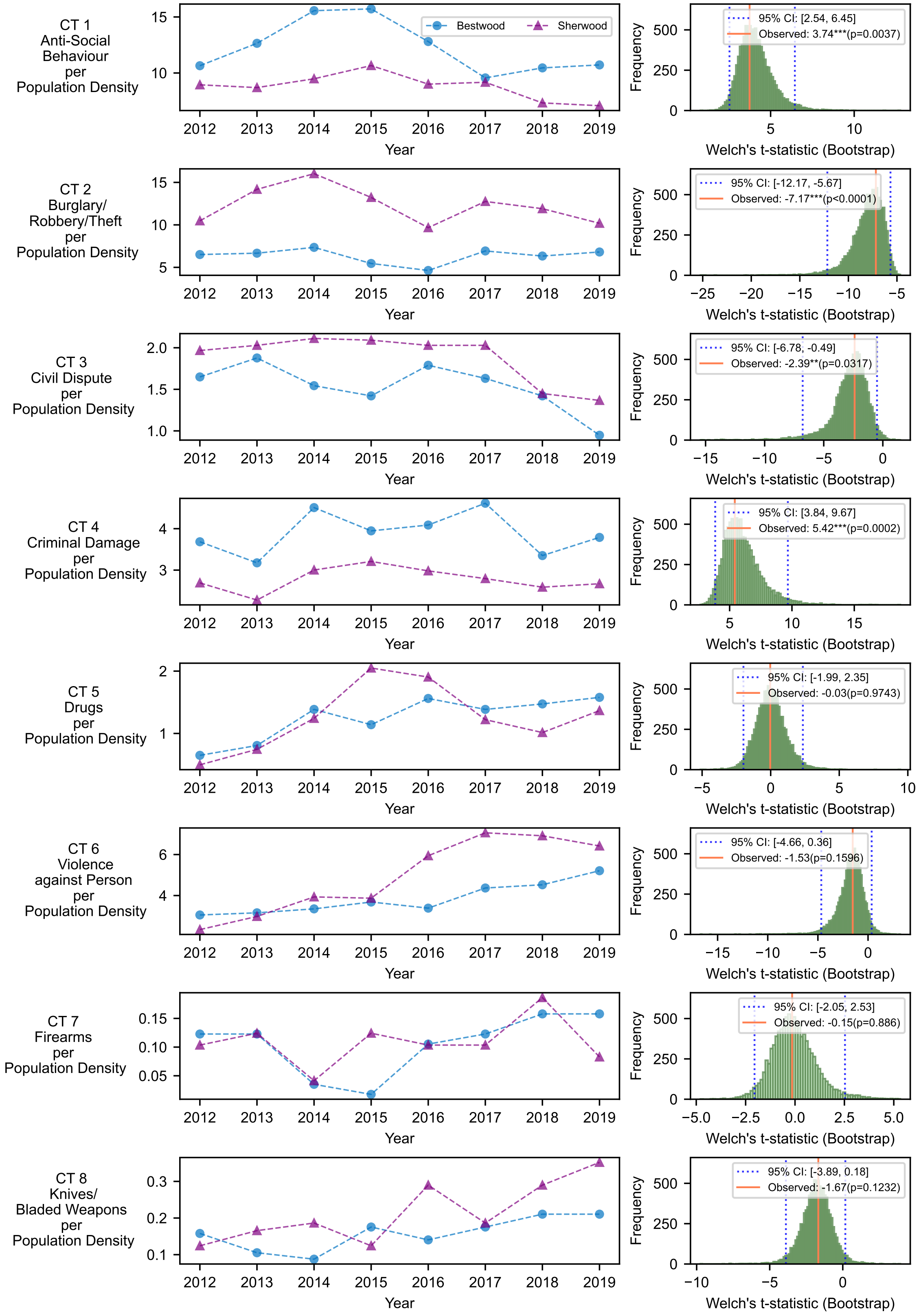

Figure S12: **Yearly crime counts per population density of eight crime types in Bestwood and Sherwood (2012–2019) and their statistical differences.** The left panel shows the observed measurements of Bestwood and Sherwood under eight CTs, respectively. The right panel shows the associated Welch's t-statistics (two-sided) from both the observed values and 10,000 bootstrapped values. $^{***}p < 0.01$, $^{**}p < 0.05$, $^{*}p < 0.1$.

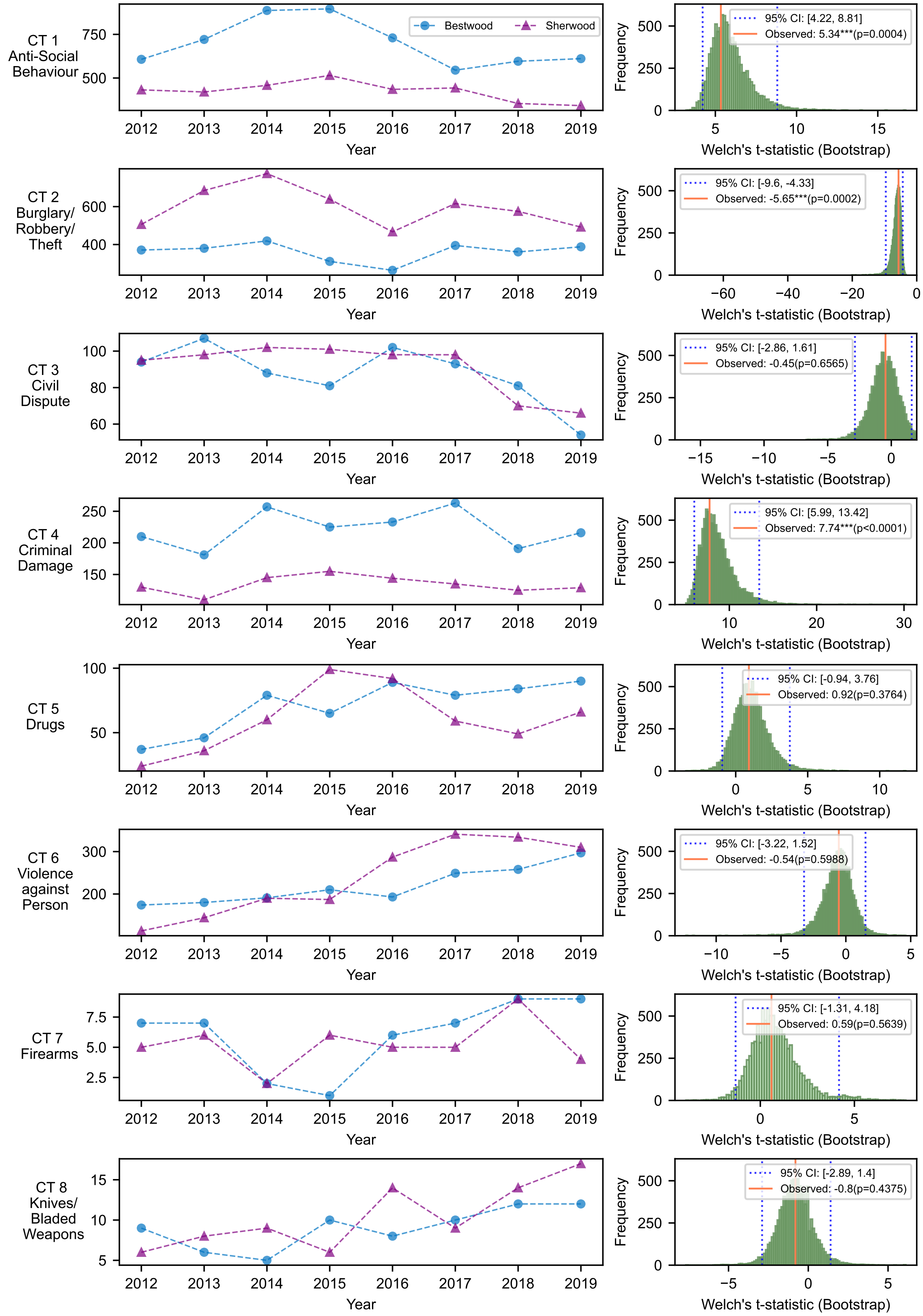

Figure S13: **Yearly crime counts of eight crime types in Bestwood and Sherwood (2012–2019) and their statistical differences.** The left panel shows the observed measurements of Bestwood and Sherwood under eight CTs, respectively. The right panel shows the associated Welch's t-statistics (two-sided) from both the observed values and 10,000 bootstrapped values.$^{***}p < 0.01$, $^{**}p < 0.05$, $^{*}p < 0.1$.

SI.6 Building environment in Nottingham city

During the implementation of this study from 2022 to 2024, we visited various places in Nottingham, including Bestwood, Bulwell, Clifton South, and the city centre. Based on our observation, we evaluate that the built environment in Nottingham is generally not untidy or messy. The town centres and residential buildings from Bestwood and Bulwell (and even from Clifton South) are generally in good shape. The typical residential buildings look similar in both areas, many built with red or brown bricks, detached or semi-detached houses. Besides, the high streets (shopping areas or town centres) between these two places do not look significantly different. The city centre of Nottingham has more commercial buildings and streets, which also look tidy.

Here, we attach a few photos of the typical street views in Bestwood and Bulwell from our trips. We also identified several city sightseeing style public videos of Bulwell ([6, 7]) and Bestwood ([8, 9]) from YouTube, which could provide a window for readers to perceive the general environment differences between these two wards visually. Additionally, we have investigated the general built environment of Bestwood and Bulwell by utilising Google Maps (https://www.google.com/maps), including its street view images, satellite maps, and default maps. We didn't recognise much ecological difference between these two wards from Google Maps either. We saw more ecological variance between Bestwood and the city centre, and between Bulwell and the city centre.

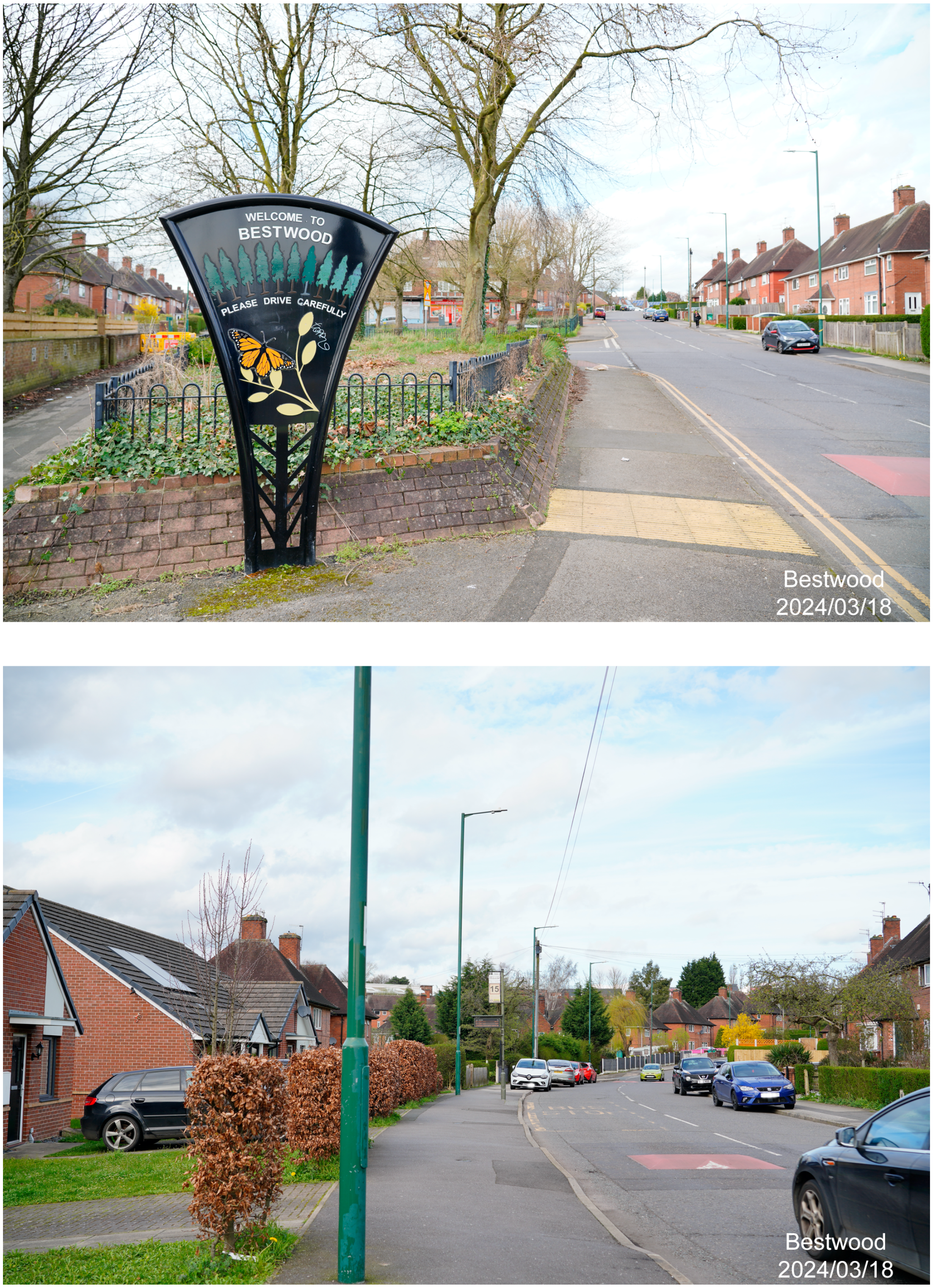

Figure S14: **Bestwood residential area.** Photos were taken in Bestwood by author F.Z., on 2024/03/18. We have masked some car plates and human faces with white pixels for privacy protection in this and the following photos.

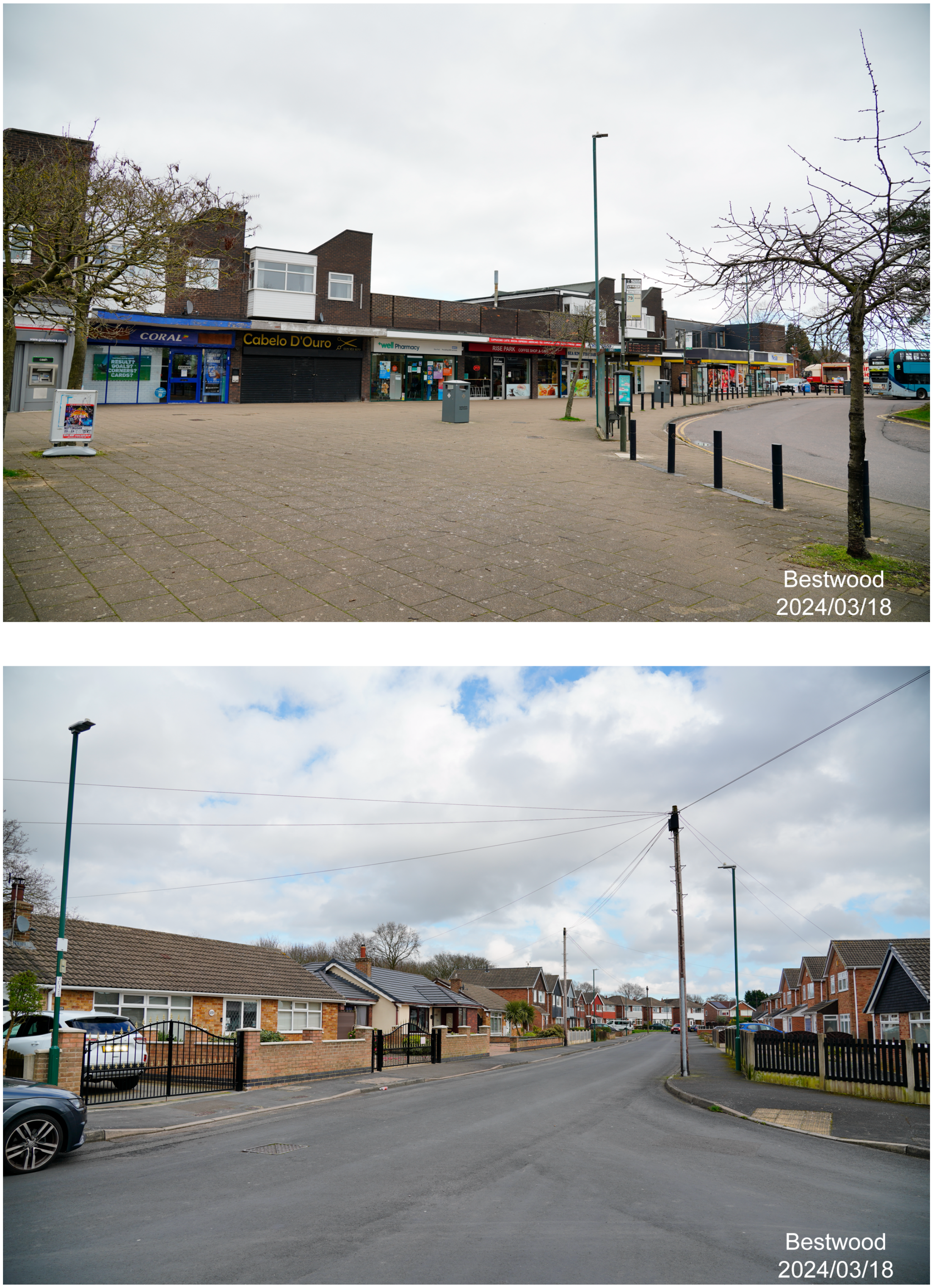

Figure S15: **Bestwood commercial and residential areas.** Photos were taken in Bestwood by author F.Z., on 2024/03/18.

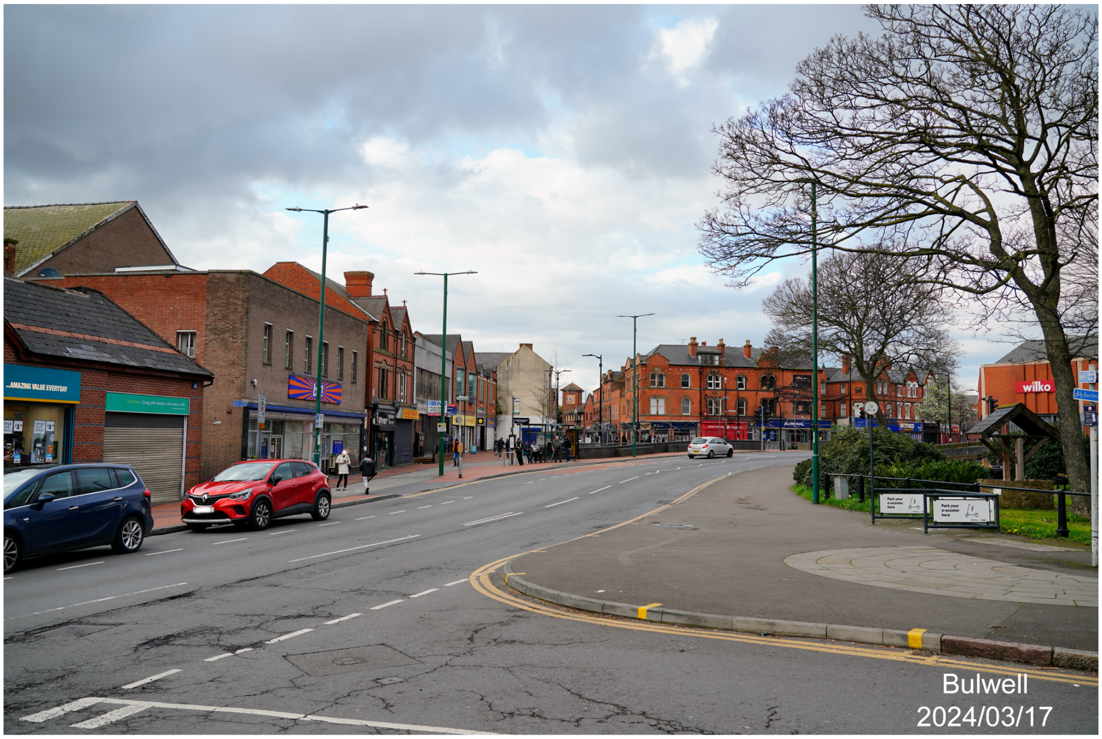

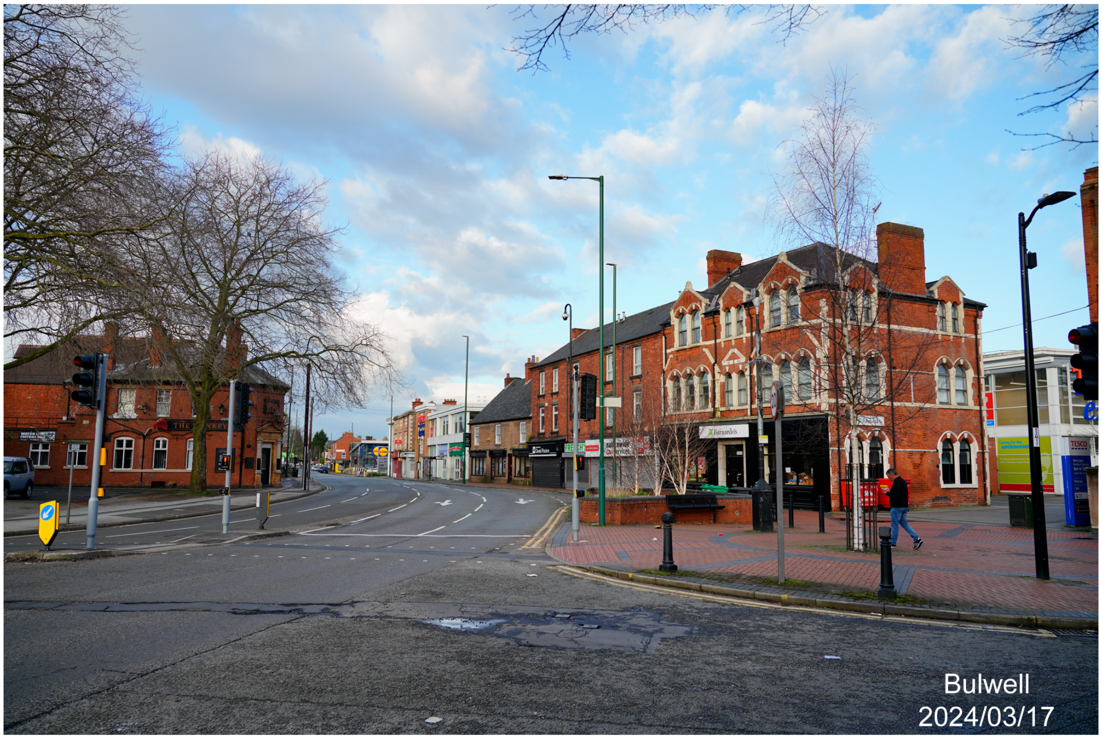

Figure S16: **Bulwell commercial area.** Photos were taken in Bulwell by author F.Z., on 2024/03/17.

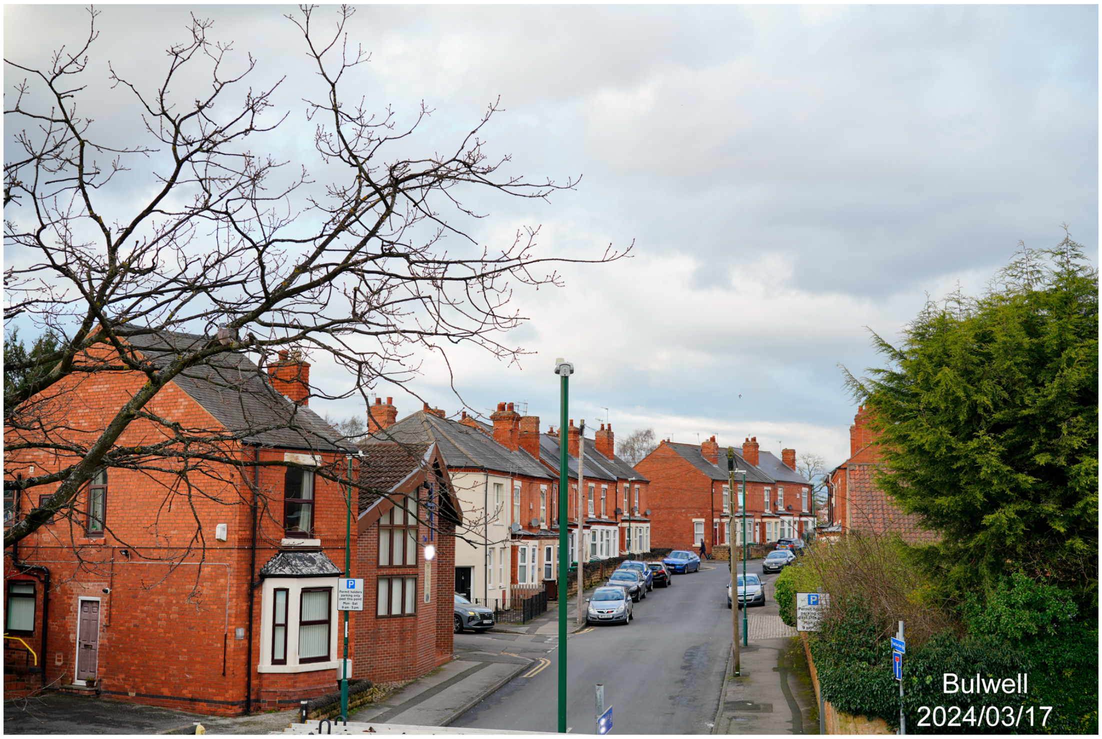

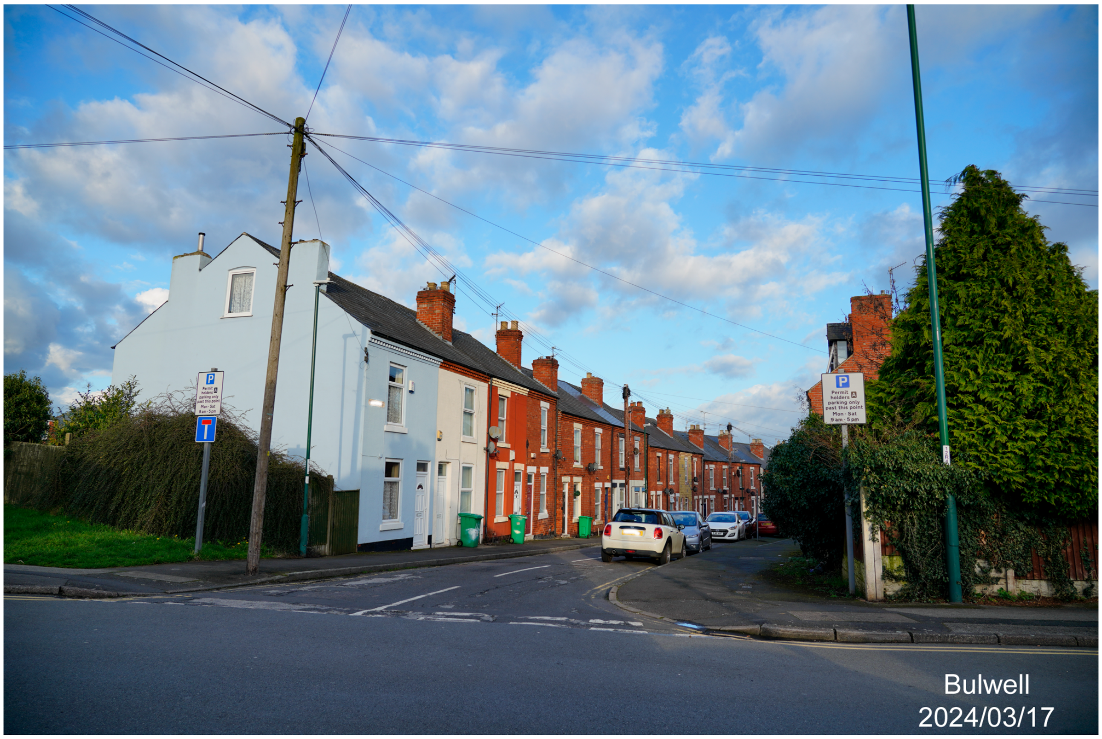

Figure S17: **Bulwell residential area.** Photos were taken in Bulwell by author F.Z., on 2024/03/17.

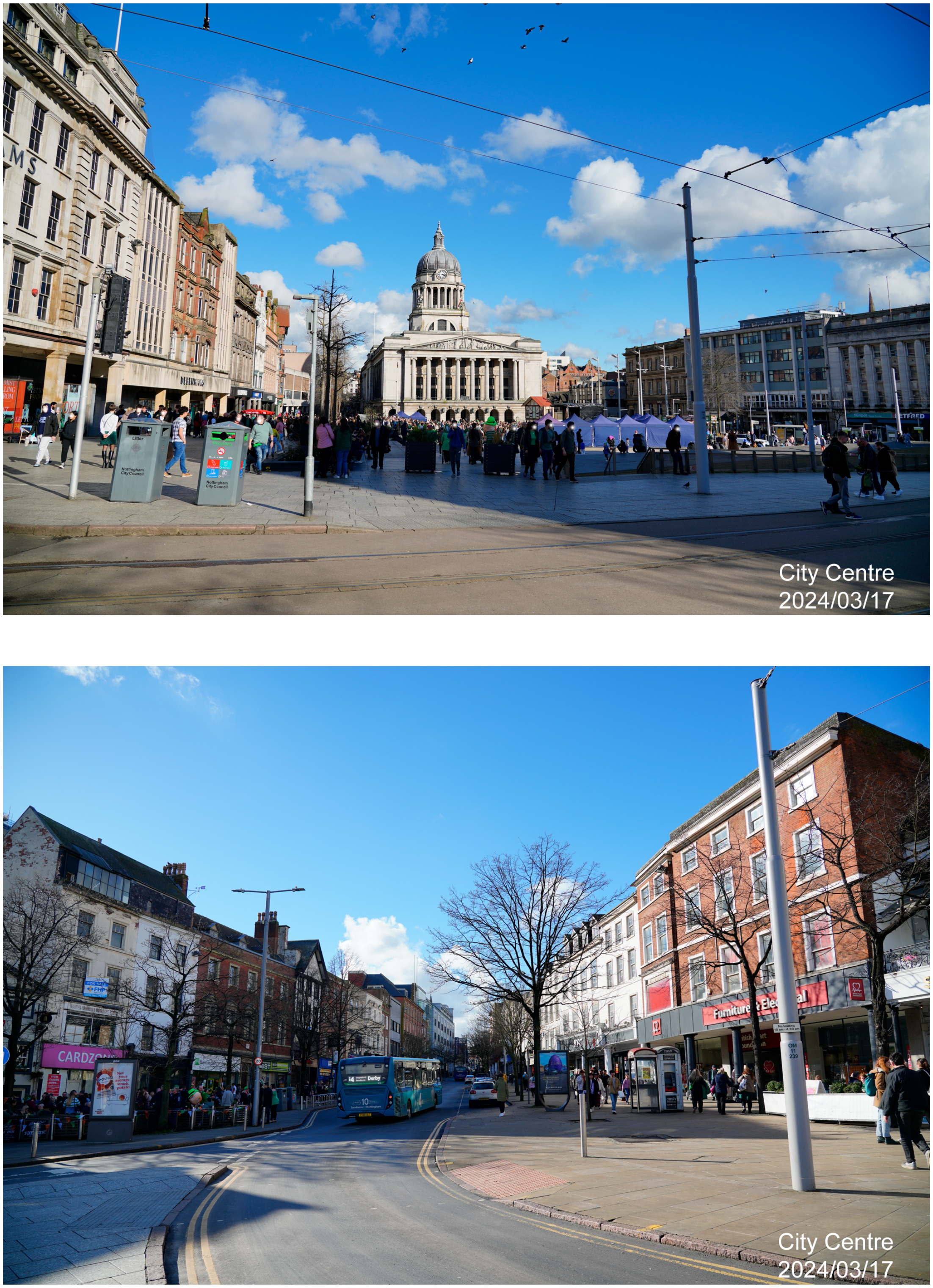

Figure S18: **City centre commercial area.** Photos were taken in Nottingham city centre by author F.Z., on 2024/03/18.

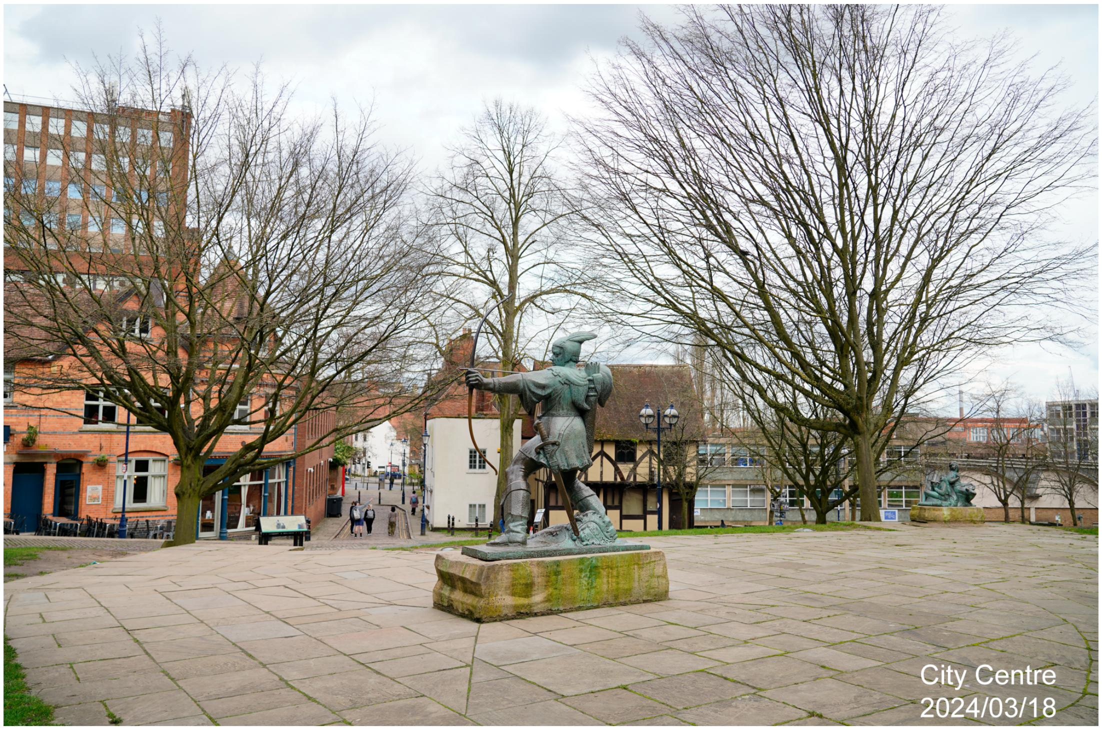

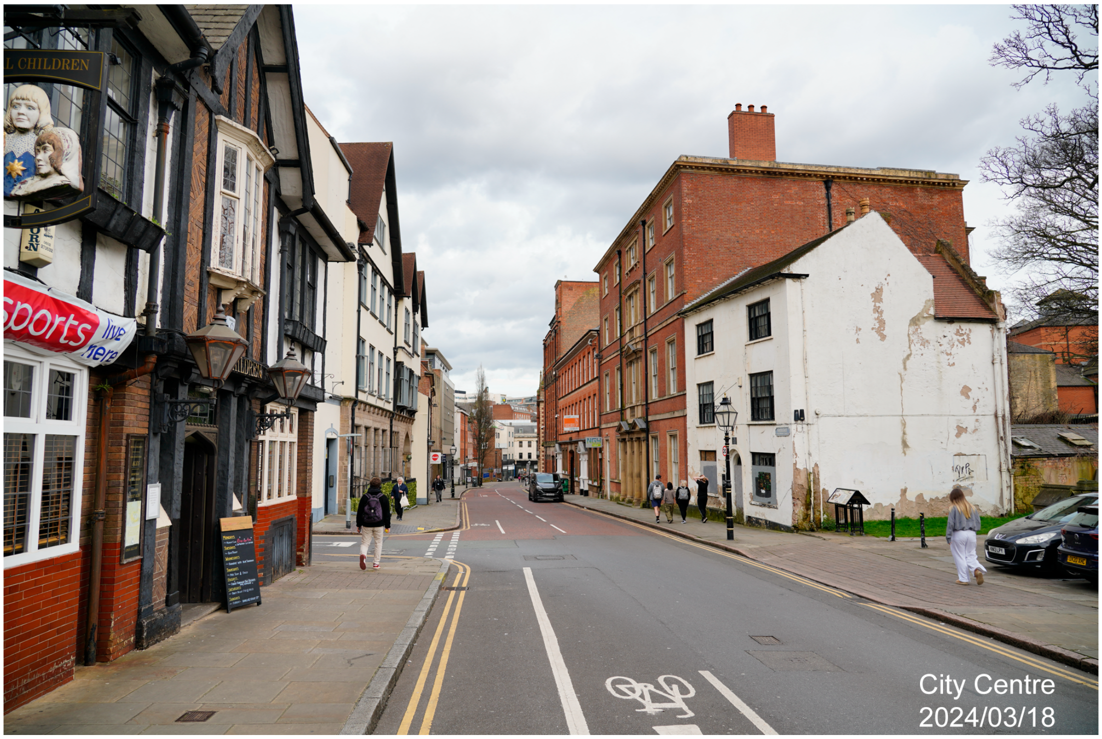

Figure S19: **City centre area.** Photos were taken in Nottingham city centre by author F.Z., on 2024/03/18.

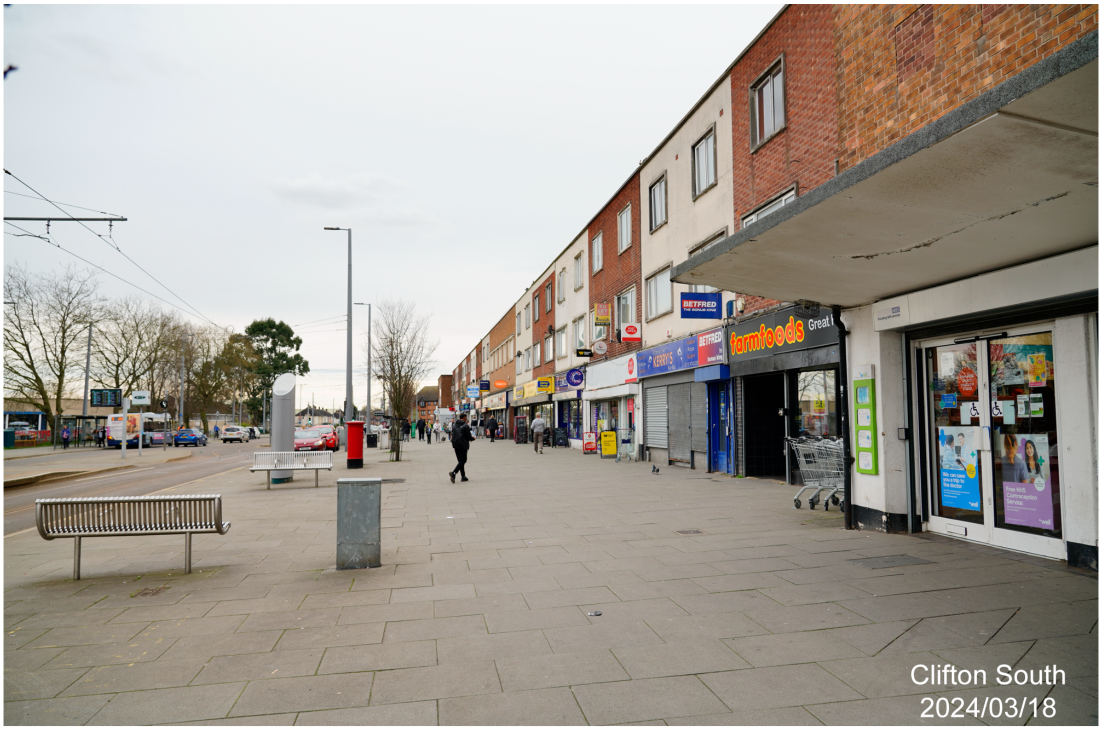

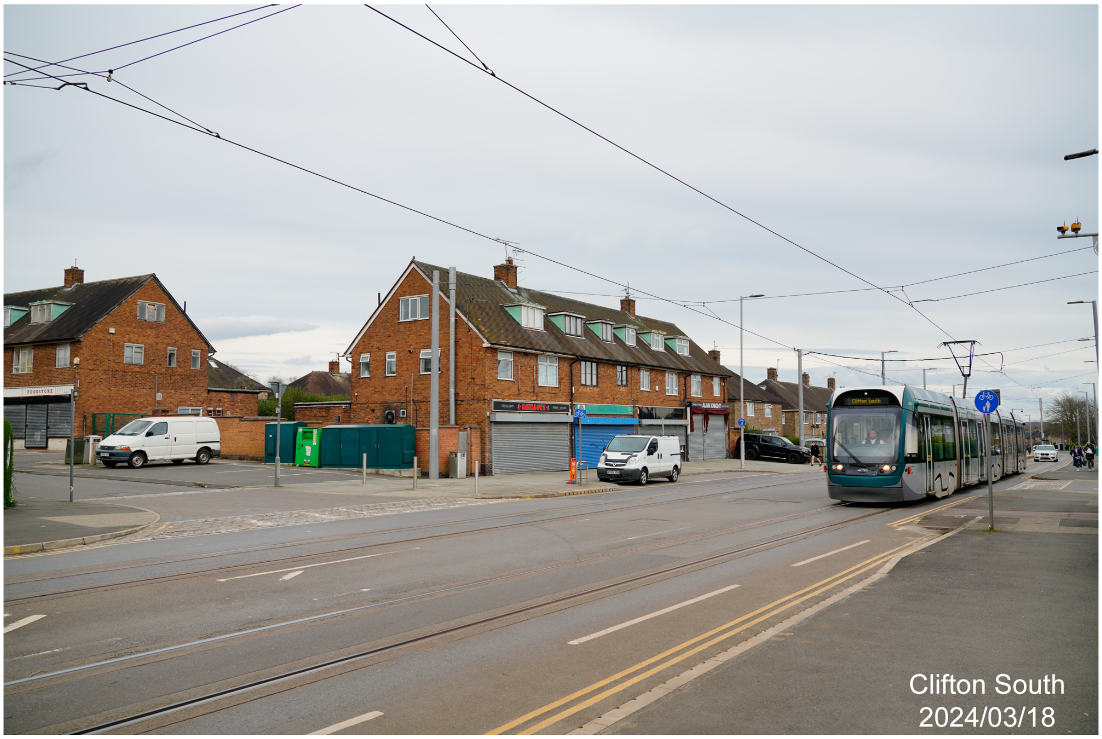

Figure S20: **Clifton South commercial area.** Photos were taken in Cliton South by author F.Z., on 2024/03/18.

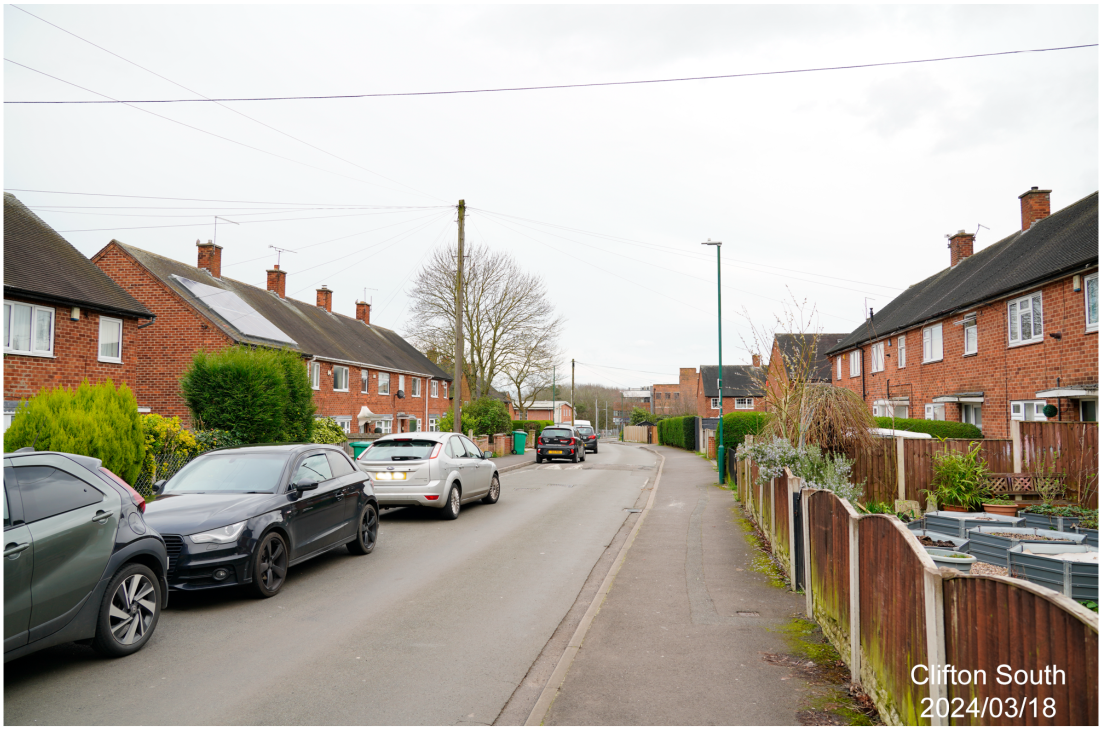

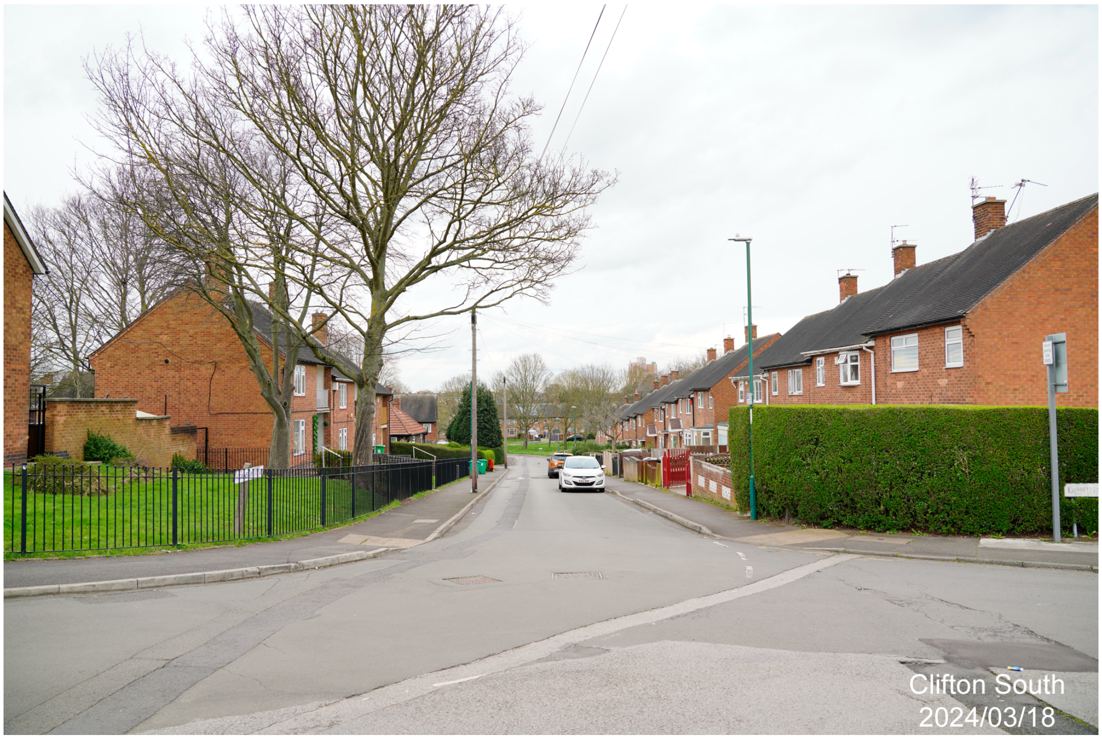

Figure S21: **Clifton South residential area.** Photos were taken in Cliton South by author F.Z., on 2024/03/18.

References

[1] Office for National Statistics. UK office for national statistics: 2011 Census data. https://www.ons.gov.uk/census/2011census. Accessed: 2023-10-30.

[2] Ministry of Housing, Communities and Local Government. English indices of deprivation 2015 - LSOA level. https://opendatacommunities.org/data/societal-wellbeing/imd/indices. Accessed: 2023-10-30.

[3] Campana, P., Varese, F. & Meneghini, C. Not all gangs are created equal: Criminal governance in London (2023). SocArXiv: osf.io/preprints/socarxiv/mt6nk.

[4] Fellstrom, C. *Hoods : The Gangs of Nottingham, A Study in Organised Crime* (Milo Books, 2010).

[5] James Sturcke. Underworld boss used Facebook to threaten enemies from jail. https://www.theguardian.com/uk/2010/jan/31/colin-gunn-facebook-threats-jail (2010). [Online; Accessed: 2024-10-09].

[6] Tour, O. Nottingham Bulwell town centre during the day. https://www.youtube.com/watch?v=kShtiDiBat8 (2024). [Online; Accessed: 2024-02-26].

[7] Tour, O. Bulwell at Nottingham in the evening time. https://www.youtube.com/watch?v=-275Y5PSRd4 (2023). [Online; Accessed: 2024-02-26].

[8] Tour, O. Bestwood Nottingham UK. https://www.youtube.com/watch?v=PY0h8zeASoc (2022). [Online; Accessed: 2024-02-26].

[9] Tour, O. Landscape photography in Bestwood Nottingham UK. https://www.youtube.com/watch?v=Dwt1co2K47A (2024). [Online; Accessed: 2024-02-26].
